# Coenzyme A protects against ferroptosis via CoAlation of mitochondrial thioredoxin reductase

**DOI:** 10.1172/JCI190215

**Published:** 2025-07-22

**Authors:** Chao-Chieh Lin, Yi-Tzu Lin, Ssu-Yu Chen, Yasaman Setayeshpour, Yubin Chen, Denise E. Dunn, Taylor Nguyen, Alexander A. Mestre, Adrija Banerjee, Lalitha Guruprasad, Erik J. Soderblom, Guo-Fang Zhang, Chen-Yong Lin, Valeriy Filonenko, Suh Young Jeong, Scott R. Floyd, Susan J. Hayflick, Ivan Gout, Jen-Tsan Chi

**Affiliations:** 1 Department of Molecular Genetics and Microbiology,; 2 Duke Center for Genomic and Computational Biology,; 3 Department of Radiation Oncology, and; 4 Department of Biochemistry, Duke University School of Medicine, Durham, North Carolina, USA.; 5 School of Chemistry, University of Hyderabad, Hyderabad, India.; 6 Proteomics and Metabolomics Core Facility,; 7 Duke Molecular Physiology Institute and Sarah W. Stedman Nutrition and Metabolism Center, and; 8 Department of Medicine, Division of Endocrinology, Metabolism, and Nutrition, Duke University School of Medicine, Durham, North Carolina, USA.; 9 Lombardi Comprehensive Cancer Center, Department of Oncology, Georgetown University, Washington, DC, USA.; 10 Department of Cell Signaling, Institute of Molecular Biology and Genetics, National Academy of Sciences of Ukraine, Kyiv, Ukraine.; 11 Department of Molecular & Medical Genetics,; 12 Pediatrics, and Neurology, Oregon Health & Science University, Portland, Oregon, USA.; 13 Department of Structural and Molecular Biology, University College London, London, United Kingdom.

**Keywords:** Cell biology, Metabolism, Amino acid metabolism, Cell stress, Mitochondria

## Abstract

The cystine-xCT transporter/glutathione/GPX4 axis is the canonical pathway protecting cells from ferroptosis. Whereas GPX4-targeting ferroptosis-inducing compounds (FINs) act independently of mitochondria, xCT-targeting FINs require mitochondrial lipid peroxidation, though the mechanism remains unclear. Because cysteine is also a precursor for coenzyme A (CoA) biosynthesis, here, we demonstrated that CoA supplementation selectively prevented ferroptosis triggered by xCT inhibition by regulating the mitochondrial thioredoxin system. Our data showed that CoA regulated the in vitro enzymatic activity of mitochondrial thioredoxin reductase-2 (TXNRD2) by covalently modifying the thiol group of cysteine (CoAlation) on Cys-483. Replacing Cys-483 with alanine on TXNRD2 abolished its enzymatic activity and ability to protect cells against ferroptosis. Targeting xCT to limit cysteine import and, therefore, CoA biosynthesis reduced CoAlation on TXNRD2. Furthermore, the fibroblasts from patients with disrupted CoA metabolism had increased mitochondrial lipid peroxidation. In organotypic brain slice cultures, inhibition of CoA biosynthesis led to an oxidized thioredoxin system, increased mitochondrial lipid peroxidation, and loss of cell viability, which were all rescued by ferrostatin-1. These findings identified CoA-mediated posttranslational modification to regulate the thioredoxin system as an alternative ferroptosis protection pathway with potential clinical relevance for patients with disrupted CoA metabolism.

## Abstract 2

Coenzyme A protects cells from ferroptosis by regulating mitochondrial thioredoxin system, revealing a new pathway with implications for genetic diseases with disrupted CoA biosynthesis.

## Graphical abstract

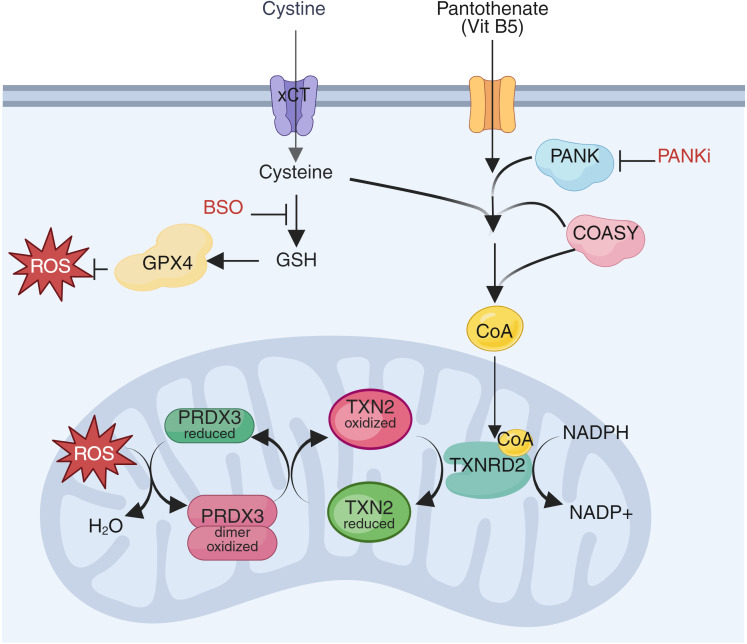

## Introduction

The de novo biosynthesis of coenzyme A (CoA) begins with the phosphorylation of extracellular pantothenate (vitamin B_5_) by pantothenate kinase (PANK), followed by the incorporation of cysteine and ATP (1). Gene mutations in enzymes involved in this pathway — particularly mitochondrial pantothenate kinase 2 (*PANK2*) and CoA synthase (*COASY*) — lead to the neurodegenerative disorders pantothenate kinase–associated neurodegeneration (PKAN) and COASY protein–associated neurodegeneration (CoPAN), respectively (2). Both PKAN and CoPAN fall under the umbrella of neurodegeneration with brain iron accumulation (NBIA), a group of inherited disorders characterized by progressive dystonia, dysarthria, spasticity, parkinsonism, and other neuropsychiatric symptoms. There currently are no effective treatments for PKAN or CoPAN, and their pathogenesis remains poorly understood (3).

CoA and its thioester derivatives are involved in diverse functions, including protein acetylation, the Krebs cycle, amino acid metabolism, fatty acid synthesis, and regulation of gene expression (4). The cytosolic CoA concentrations are estimated in the micromolar range, whereas mitochondrial CoA levels are substantially higher, up to millimolar concentrations, suggesting a particularly crucial role within mitochondria (5). A recently discovered function of CoA involves its covalent attachment to cysteine residues in proteins under oxidative stress, a posttranslational modification (PTM) termed “CoAlation” (6). More than 2,000 mammalian and bacterial proteins have been identified to be CoAlated (6), yet the regulation and functional consequences of this modification remain largely unclear (7).

In pancreatic tumors, CoA has been proposed to support ferroptosis resistance by enhancing coenzyme Q10 (CoQ10) production via the mevalonate pathway (8). Ferroptosis is a regulated form of cell death characterized by iron dependency, oxidative stress, and lipid peroxidation. Most mammalian cells have multiple endogenous ferroptosis-protecting mechanisms. Key among these is the glutathione-dependent antioxidant defense, whereby glutathione peroxidase 4 (GPX4) reduces lipid hydroperoxides, thus preventing membrane damage and ferroptosis. The xCT antiporter plays a critical role by importing cystine (the dimeric form of cysteine) for glutathione (GSH) synthesis, which is essential for GPX4 function. Additionally, ferroptosis suppression protein 1 (FSP1) provides a GPX4-independent defense by regenerating CoQ10, a lipophilic radical-trapping antioxidant that inhibits lipid peroxidation (9). Therefore, targeting these ferroptosis protection proteins with various ferroptosis-inducing compounds (FINs) may trigger ferroptosis.

FINs can be categorized into different classes based on their targets and mechanisms. Class I FINs (e.g., erastin, sulfasalazine) inhibit the xCT transporter, blocking cystine uptake and depleting GSH. Class II FINs (e.g., RSL3, ML162) directly inhibit GPX4. Class III FINs deplete both GPX4 and CoQ10 (10). Notably, although both class I and class II FINs impair the xCT/GSH/GPX4 axis, only class I FINs are associated with mitochondrial lipid peroxidation (11, 12). Although the requirement of mitochondria for class I FIN–induced ferroptosis has been established, the molecular mechanism remains unclear (11).

The thioredoxin (TXN) system operates in parallel with the GSH system to maintain redox balance and suppress ferroptosis (13). The TXN system facilitates the sequential transfer of electrons from NADPH to thioredoxin reductase (TXNRD), then to TXN, and finally to peroxiredoxins (PRDX), enabling the elimination of intracellular ROS. In mammalian cells, the TXN system is compartmentalized: the cytosolic system consists of TXN1, TXNRD1, and PRDX1, and the mitochondrial system includes TXN2, TXNRD2, and the mitochondria-specific peroxiredoxin (PRDX3).

Buthionine sulfoximine (BSO) inhibits GSH biosynthesis by targeting γ-glutamylcysteine synthetase but is a much weaker FIN than erastin. In a study, researchers proposed that GSH depletion by BSO leads to cystine accumulation, which enhances the TXN system to counteract ferroptosis (14). This could explain why combining BSO with TXNRD2 inhibitors, such as auranofin, leads to synergistic cell death (14). Nevertheless, the precise mechanism underlying this interplay remains to be elucidated.

In the present study, we found that CoA effectively prevents class I FIN–induced ferroptosis. CoA availability modulates the redox state of the mitochondrial TXN system, suppressing mitochondrial lipid peroxidation and thereby protecting cells from ferroptosis. Mechanistically, CoA covalently modifies Cys-483 of TXNRD2 via CoAlation, enhancing its enzymatic activity and redox capacity during ferroptotic stress. Consistently, fibroblasts from patients with PKAN also had increased mitochondrial lipid peroxidation. These findings were validated in organotypic brain slice cultures (OBSCs), an ex vivo model that preserves cytoarchitecture and neuron-glia interactions. Our work uncovers a class I FIN–specific requirement for mitochondrial lipid peroxidation and identifies cysteine-derived CoA as a critical regulator of TXN redox signaling. These insights may have clinical relevance for PKAN, CoPAN, and other disorders involving CoA metabolism.

## Results

### CoA specifically protected against ferroptosis induced by xCT inhibitors.

**Figure 1:**
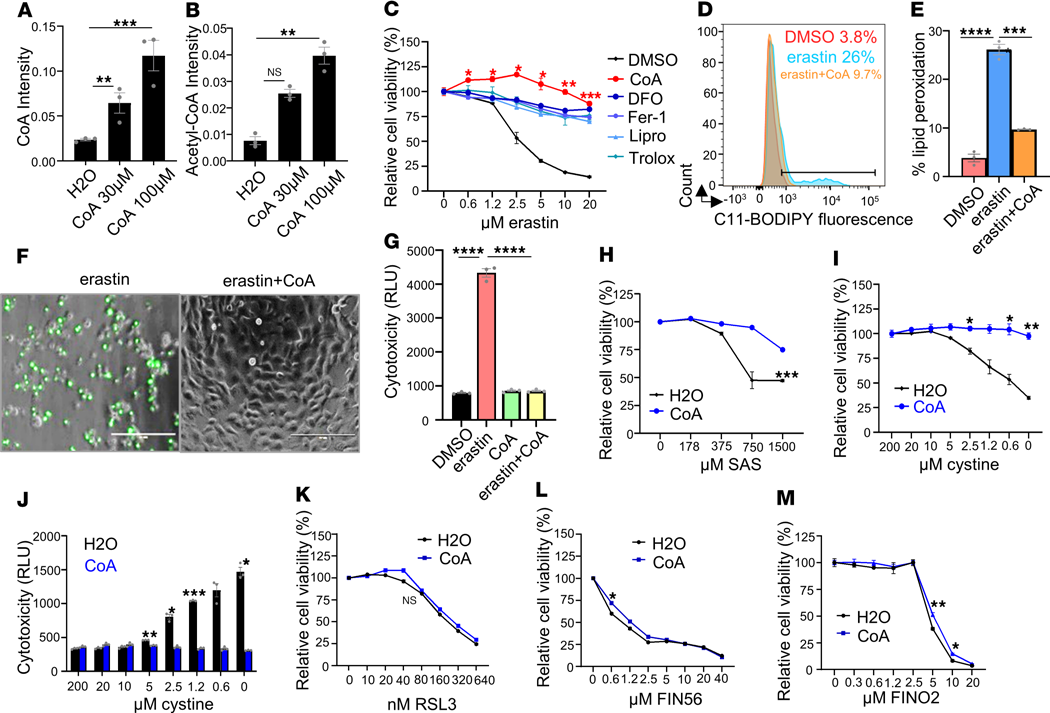
CoA is a specific ferroptosis inhibitor against xCT inhibitors. (**A** and **B**) CoA supplementation increased the levels of intracellular CoA (**A**) and acetyl-CoA (**B**) as quantified by LC-MS/MS analysis. HT-1080 cells were treated with H_2_O and 2 concentrations of CoA (30 μM and 100 μM) for 18 hours for LC-MS/MS analysis. (**C**) CoA inhibited erastin-induced ferroptosis. HT-1080 cells were treated with increasing doses of erastin, either alone or in combination with CoA (100 μM) or deferoxamine (80 μM), Fer-1 (10 μM), lipro (2 μM), or Trolox (100 μM). The cell viability was quantified by Cell-Titer Glo assay. (**D** and **E**) Erastin-induced lipid peroxidation (2 μM, 18 hours) in HT-1080 cells was inhibited by CoA treatment as determined by C11-BODIPY staining (**D**) and the quantification of the percentage of lipid peroxidation–positive cells (**E**). (**F** and **G**) CoA (100 μM) inhibited erastin-induced membrane rupture (erastin: 2.5 μM, 20 hours) in HT-1080 cells, as observed by CellTox Green under fluorescence microscope (**F**) and quantified by a plate reader (**G**). (**H**) CoA (100 μM) inhibited class I FIN–induced ferroptosis (via (sulfasalazine [SAS], 20 hours) in HT-1080 cells. (**I** and **J**) CoA (100 μM) inhibited 24 hours of cystine deprivation-induced ferroptosis as determined by Cell-Titer Glo assay (**I**) or CellTox Green assay (**J**). (**K**–**M**) CoA (100 μM) did not inhibit ferroptosis in HT-1080 cells induced by RSL3 (class II FIN, 20 hours) (**K**), FIN56 (class III FIN, 20 hours), and FINO2 (class IV FIN, 20 hours) (**M**). (**A**, **B**, **E**, and **G**) Data were analyzed by 1-way ANOVA and Tukey’s multiple comparisons. (**C** and **H**–**M**) Data were analyzed by 2-way ANOVA and Šídák’s multiple comparisons; *n* = 3 independent biological replicates. *P < 0.05, **P < 0.01, ***P < 0.001, and ****P < 0.0001. Data represent mean ± SEM.

Supplementation of CoA in culture medium protects mouse embryonic fibroblasts and pancreatic tumors from ferroptosis (8, 15). Given no known plasma membrane transporter was found, CoA was proposed to be degraded to pantothenate (16), or 4′-phosphopantetheine (4′-PPT) (17) to increase intracellular CoA. Although the detailed mechanism of CoA import remains controversial, we supplemented regular HT-1080 cell culture medium with 2 concentrations of CoA (30 μM and 100 μM) for 18 hours. In previous studies 200 μM (8) and 500 μM (14) CoA supplement were used to rescue ferroptosis; however, we found in the present study that relatively lower doses of CoA supplement (30 μM and 100 μM) were sufficient to increase the intracellular levels of CoA (Figure 1A) and acetyl-CoA (Figure 1B) as measured by mass spectrometry (MS). The increase in acetyl-CoA levels further confirmed that the supplemented CoA could serve as an acetyl group carrier (Figure 1B).

Next, we measured the protective capacity of CoA and several canonical ferroptosis inhibitors, including deferoxamine, ferrostatin-1 (Fer-1), liproxstatin-1 (lipro), and Trolox, against erastin-induced ferroptosis in HT-1080 cells. We found that the ferroptosis protective effect of CoA was comparable to those of the canonical ferroptosis inhibitors (Figure 1C). Subsequently, we further confirmed that CoA also significantly protected against the erastin-induced ferroptosis in 6 cancer cell lines (HEK-293, 786-O, H1975, RCC4, MDA-MB-231, and IGROV-1 cells) (Supplemental Figure 1, A–F; supplemental material available online with this article; https://doi.org/10.1172/JCI190215DS1). Consistently, CoA also abolished other molecular features of erastin-induced ferroptosis, including lipid peroxidation (flow cytometry data are presented in Figure 1, D and E) and membrane rupture (CellTox Green data are shown in Figure 1, F and G). Moreover, we found that CoA inhibited ferroptosis triggered by different class I FINs with distinct chemical structures (erastin, sulfasalazine, and BSO) (Figure 1, C and H, and Supplemental Figure 1G) or cystine deprivation (Figure 1, I and J). However, CoA had only modest or no protective effects against ferroptosis induced by 3 chemically distinct class II FINs (RSL3, ML162, and JKE-1674) (Figure 1K and Supplemental Figure 1, H and I), a class III FIN (FIN56) (Figure 1L), or a class IV FIN (FINO2) (Figure 1M). Notably, the effects of CoA on various chemically distinct FINs and cystine deprivation largely excluded the potential off-target effects of CoA on any particular FINs.

Interestingly, ionizing radiation and related pathways have been reported to regulate ferroptosis (18–20). We found that CoA also rescued radiation-induced cell death (Supplemental Figure 1J). Taken together, these results indicate that CoA-mediated inhibition of ferroptosis may regulate ferroptosis processes upstream of GPX4, which is targeted by various class II and III FINs.

### The mitochondrial TXN system determines CoA-mediated ferroptosis inhibition.

To uncover the underlying mechanisms of CoA-mediated ferroptosis inhibition, we performed a target compound screen to identify compounds that could abolish CoA-mediated ferroptosis protection. Previously, CoA was proposed to inhibit lipid peroxidation of pancreatic cancer cells by synthesizing CoQ10 for FSP1 via the mevalonate pathway (8). Thus, we first determined whether the CoA-protected ferroptosis could be abolished by the FSP1 inhibitor FSEN1 (Supplemental Figure 2A). Although FSEN1 was able to sensitize ferroptosis in the control cells, FSEN1 did not abolish CoA-mediated inhibition of ferroptosis in HT-1080 cells (Supplemental Figure 2A).

Next, we evaluated the efficacy of various inhibitors targeting pathways recognized to be critical for ferroptosis protection to determine their potential to abolish CoA-mediated ferroptosis protection. These inhibitors included brequinar and teriflunomide (12), which are dihydroorotate dehydrogenase inhibitors (Supplemental Figure 2, B and C); etomoxir, a β-oxidation inhibitor (21) (Supplemental Figure 2D); methotrexate, a dihydrofolate reductase inhibitor (22) (Supplemental Figure 2E); compound C, an AMPK inhibitor (23) (Supplemental Figure 2F); 5-tetradecyloxy-2-furoic acid (TOFA), an acetyl-CoA carboxylase (24) (Supplemental Figure 2G); and the inhibitors of the *S*-adenosyl homocysteine hydrolase of the trans-sulfuration pathway (25) (Supplemental Figure 2H). However, none of these compounds mitigated the ferroptosis protection effect of CoA (Supplemental Figure 2, A–H).

Given that ACSL3 mediates the incorporation of CoA into monounsaturated fatty acids (MUFAs) (26, 27), we conducted a genetic knockdown of ACSL3 and found that it had no effect on CoA-mediated protection against ferroptosis (Supplemental Figure 2, I and J).

**Figure 2:**
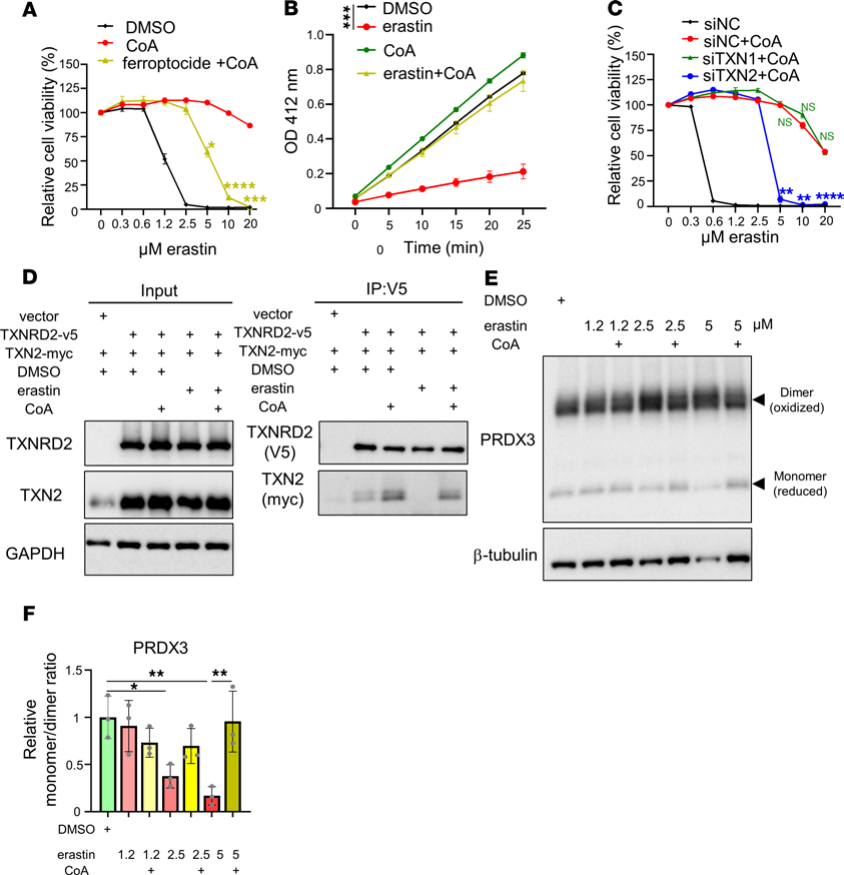
CoA regulates mitochondrial TXN system. (**A**) HT-1080 cells were treated with CoA (100 μM) alone or in combination with the TXN inhibitor ferroptocide (2 μM), during erastin-induced ferroptosis (20 hours). Cell viability was quantified using the Cell-Titer Glo assay. (**B**) TXNRD activity was significantly reduced after erastin treatment (1.25 μM, 16 hours) in HT-1080 cell lysates, and this repression was restored by CoA (100 μM) supplementation. (**C**) Pooled siRNA knockdown of TXN2, but not TXN1, abolished CoA-mediated protection from ferroptosis, indicating a specific role for mitochondrial TXN2. siNC, negative control siRNA. (**D**) Erastin disrupted the interaction between TXNRD2 and TXN2, which was restored by CoA. HT-1080 cells overexpressing TXNRD2 and TXN2 were treated with erastin (2.5 μM, 18 hours), with or without CoA, and lysed in NEM buffer for coimmunoprecipitation analysis. (**E**) Western blot analysis revealed that erastin reduced the levels of PRDX3 monomers (reduced, active forms), which was reversed by CoA supplementation. (**F**) Monomer/dimer ratios of PRDX3 were quantified in cells treated with erastin and CoA. Data were analyzed by 1-way ANOVA and Tukey’s test. (**A**–**C**) Data were analyzed by 2-way ANOVA and Šídák’s test; *n* = 3 biological replicates. Data are reported as mean ± SEM. *P < 0.05, **P < 0.01, ***P < 0.001, and ****P < 0.0001.

Among the compounds tested, we found that ferroptocide (28) (a TXN inhibitor) abolished CoA-mediated inhibition of ferroptosis (Figure 2A). Furthermore, CoA could not rescue ferroptocide-induced cell death (Supplemental Figure 2K). These data suggest the TXN system is critical in the CoA-mediated inhibition of ferroptosis.

Although any screens may have false-negative results, and our results did not completely rule out the involvement of other pathways, the ability of ferroptocide prompted us to further investigate the TXN pathways. Ferroptocide triggers ferroptosis by inhibiting both the cytosolic and mitochondrial versions of TXN (28). Given TXN and TXNRD2 are both required for a functional TXN system, we examined TXN and TXNRD2 activities in HT-1080 cell lysates when they were treated with erastin, CoA, and their combination (Figure 2B and Supplemental Figure 2L). Although TXN activity in HT-1080 cell lysates was not altered by erastin or CoA (Supplemental Figure 2L), erastin significantly reduced TXNRD activity, which was restored by CoA supplementation (Figure 2B). However, the protein levels of both cytosolic and mitochondrial TXNRDs (namely, TXNRD1 and TXNRD2) were not affected by erastin or CoA (Supplemental Figure 2M). Because erastin reduces the intracellular levels of CoA, we speculated that reduced CoA levels may regulate TXNRD activity during ferroptosis.

To determine the relative importance of cytosolic versus mitochondrial TXN systems for CoA-mediated inhibition of ferroptosis, we used pooled siRNAs against cytosolic thioredoxin 1 (TXN1) and mitochondrial thioredoxin 2 (TXN2) to knock down these 2 genes individually (Supplemental Figure 2N). The knockdown of TXN2, but not TXN1, abolished CoA-mediated ferroptosis inhibition (Figure 2C), and this result was further validated using individual TXN2 siRNAs (Supplemental Figure 2O). These data indicate that the mitochondrial TXN system is required for CoA-mediated protection from ferroptosis. To address the concern that siRNA treatment may lead to ferroptosis sensitization in a nontarget-dependent manner (29), we conducted CRISPR knockout of TXN2 and found similar abolishment of CoA-mediated ferroptosis inhibition (Supplemental Figure 2P).

Previous findings suggested that unused cysteine directly fuels the TXN system to prevent BSO-induced ferroptosis (14); however, we hypothesized that unused cysteine is converted to CoA to regulate ferroptosis. The TXN system transfers electrons in the order of NADPH, TXNRD, TXN, and PRDXs to neutralize intracellular ROS. Given the importance of mitochondrial TXN2 (Figure 2C), we focused on the effects of erastin on the mitochondrial TXN system and examined the protein-protein interaction between TXNRD2 and its substrate TXN2. Although TXNRD2 knockout did not alter TXN1 or TXN2 levels (Supplemental Figure 2Q), through coimmunoprecipitation, we found that erastin treatment abolished the interaction between TXNRD2 and TXN2, whereas CoA supplementation rescued this interaction (Figure 2D). The redox status of the mitochondrial TXN system can be monitored by the monomer (reduced and active)/dimer (oxidized and inactive) ratio of PRDX3 (30). Thus, we treated HT-1080 cells with erastin alone or combined with CoA to measure the monomer/dimer ratio of PRDX3 (Figure 2, E and F). We noticed that erastin decreased the monomer (reduced and active form) of mitochondrial PRDX3 (Figure 2, E and F), which was rescued by CoA supplementation (Figure 2, E and F). These results suggest that CoA supplement may inhibit ferroptosis by maintaining the redox function of mitochondrial TXN system.

### Simultaneous inhibition of GSH and CoA synthesis results in synthetic lethality.

**Figure 3:**
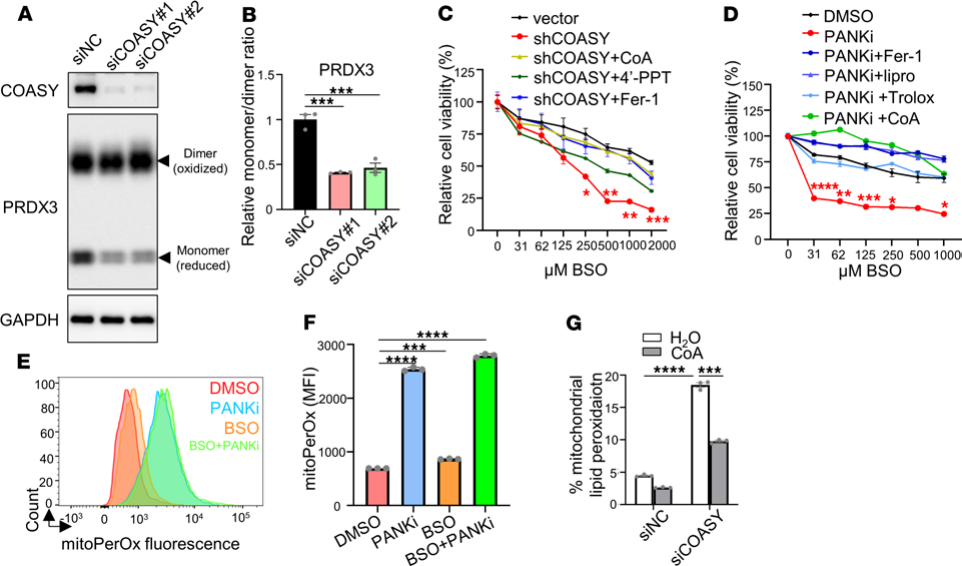
Combined inhibition of GSH and CoA synthesis leads to synthetic lethality. (**A**) Knockdown of COASY using 2 independent siRNAs decreased PRDX3 monomer levels, further linking mitochondrial redox regulation to CoA biosynthesis. (**B**) Quantification of PRDX3 monomer/dimer ratios upon COASY knockdown. siNC, negative control siRNA. (**C**) Stable COASY knockdown sensitized HT-1080 cells to BSO-induced ferroptosis, which was rescued by CoA (100 μM), 4′-PPT (100 μM), or Fer-1 (10 μM). (**D**) CoA inhibited ferroptosis induced by BSO and PANKi cotreatment. HT-1080 cells were exposed to PANKi (2.5 μM) and increasing doses of BSO with Fer-1, lipro (2 μM), Trolox (100 μM), or CoA (100 μM). (**E** and **F**) PANKi, but not BSO, increased mitochondrial lipid peroxidation, visualized with mitoPerOx (**E**) and quantified by mean fluorescence intensity (**F**). (**G**) COASY knockdown increased mitochondrial lipid peroxidation, which was rescued by CoA. (**B** and **F**) Data were analyzed by 1-way ANOVA and Tukey’s test. (**C**, **D**, and **G**) Data were analyzed by 2-way ANOVA and Šídák’s test; *n* = 3 biological replicates. Data are mean ± SEM. *P < 0.05, **P < 0.01, ***P < 0.001, and ****P < 0.0001.

Our previous findings showed that the knockdown of COASY reduced intracellular CoA levels (31). Therefore, we reduced CoA levels by knocking down COASY and assessed the redox status of the mitochondrial TXN system by measuring the monomer/dimer ratio of PRDX3 (Figure 3A). Consistently, COASY knockdown decreased the reduced form (monomer) of PRDX3 (Figure 3B). This reduced monomer/dimer ratio of PRDX3 suggests a defect in mitochondrial TXN function upon erastin treatment (Figure 2, E and F) and COASY knockdown (Figure 3, A and B).

These results prompted us to hypothesize that GSH and CoA may mediate parallel pathways of ferroptosis protection. To determine the contribution of the erastin-depleted GSH and CoA on ferroptosis, we combined BSO (a GSH inhibitor) and COASY knockdown (Figure 3C). Although BSO itself is a much less potent inducer of ferroptosis, we found that combining BSO with COASY knockdown significantly decreased cell viability over extended incubation, and this was rescued by supplementing CoA or Fer-1 (Figure 3C and Supplemental Figure 3A).

The compound 4′-PPT is an endogenous intermediate metabolite in the CoA biosynthesis pathway and is currently being evaluated as a treatment for PKAN (32). We found that 4′-PPT could also rescue the cell death caused by the BSO and COASY knockdown (Figure 3C). Similarly, a previous report showed that combining BSO and a PANK inhibitor (PANKi), a CoA biosynthesis inhibitor (33), also triggers ferroptosis in pancreatic tumors (8). Therefore, we combined BSO and PANKi and found this combination led to dramatic cell death in HT-1080 cells, which was rescued by CoA, ferroptosis inhibitors (Fer-1, lipro, and Trolox), and mitoTEMPO (34), a mitochondria-targeted antioxidant (Figure 3D and Supplemental Figure 3B). To eliminate the potential off-target effect of BSO, we knocked out the catalytic subunit of GCL (GCLC) and consistently found it also triggered cell death when combined with pharmacological inhibition with PANKi (Supplemental Figure 3C) or genetic inhibition with siCOASY (Supplemental Figure 3D) of CoA biosynthesis.

Given that COASY knockdown led to impaired redox status of the mitochondrial TXN system and the relevance of the mitochondrial TXN system for CoA-mediated ferroptosis inhibition (Figure 2C and Figure 3A), we treated HT-1080 cells with PANKi, BSO, or a combination of both. Inhibition of CoA synthesis by PANKi, compared with DMSO and BSO, indeed led to robust mitochondrial lipid peroxidation (Figure 3, E and F) and overall lipid peroxidation (Supplemental Figure 3, E and F). Consistently, genetic inhibition of CoA synthesis by COASY siRNA significantly increased mitochondrial lipid peroxidation, which could be abolished by CoA supplementation (Figure 3G). Taken together, although previous findings suggested cystine/cysteine directly fuels the TXN system to prevent BSO-induced ferroptosis (14), our data indicate cysteine incorporation in CoA biosynthesis is essential for regulating mitochondrial TXN system to protect against ferroptosis.

### CoA supplement increased CoAlation on TXNRD2 and TXNRD2-TXN2 interaction.

**Figure 4:**
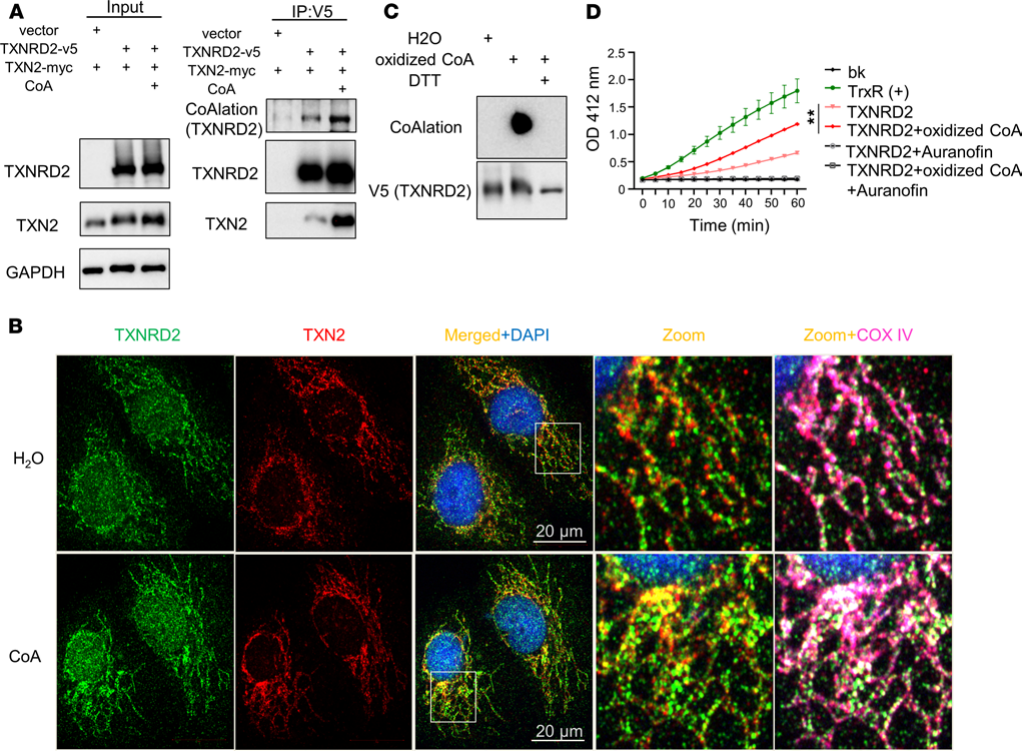
CoA supplement increased CoAlation of TXNRD2 and its interaction with TXN2. (**A**) HT-1080 cells were cotransfected with expression vectors for TXNRD2 (V5-tagged) and TXN2 (Myc-tagged), followed by treatment with CoA (100 μM) for 18 hours. TXNRD2 was immunoprecipitated using anti-V5 beads and analyzed by nonreducing SDS-PAGE. CoAlation was detected with a pan-CoAlation antibody, showing enhanced conjugation upon CoA supplementation. Interaction between TXNRD2 and TXN2 was assessed by probing the V5 pulldown for TXN2 using an anti-Myc antibody. CoA treatment increased TXN2 association, suggesting CoAlation promotes TXNRD2-TXN2 complex formation. (**B**) Confocal microscopy showed that CoA supplement increased TXNRD2-TXN2 interaction in mitochondria. COX IV is a mitochondria marker. (**C**) Specificity of CoAlation detection was validated by DTT treatment, which abolished the signal. V5-purified TXNRD2 was incubated with oxidized CoA ± DTT, analyzed by nonreducing PAGE, and immunoblotted. (**D**) CoAlation enhanced TXNRD activity. Activity was measured using purified TXNRD2 with or without CoAlation or TXNRD inhibitor (auranofin), alongside background (bk) and TrxR(+) control. Data were analyzed by 2-way ANOVA and Šídák’s test; *n* = 3 biological replicates. **P < 0.01. Data presented as mean ± SEM.

Although the protein levels of TXNRD remained unchanged, the enzymatic activity of TXNRD was significantly inhibited by erastin, and such inhibition could be rescued by CoA, suggesting an involvement of PTMs (Figure 2B and Supplemental Figure 2M). Recently, CoA has been demonstrated to modify proteins by covalent attachment (CoAlation) to the thiol groups of cysteine residues of various cellular proteins, thereby regulating their enzymatic activities (6). Interestingly, mitochondrial TXNRD2 is 1 of the potential CoAlated hits (6). Thus, we raised an mAb that specifically recognized CoA and CoAlated proteins (Supplemental Figure 4A). By supplementing CoA in HT-1080 cells overexpressing TXNRD2-V5 and TXN2-myc, we purified TXNRD2 protein by V5 agarose and blotted TXNRD2 protein with CoAlation antibody (Figure 4A). Indeed, TXNRD2 protein showed an endogenous level of CoAlation, which was further enhanced by CoA supplementation (Figure 4A). We also found that the TXN2 protein coimmunoprecipitated with TXNRD2 purification also increased with CoA supplement, suggesting that CoA supplement increases the TXNRD2-TXN2 interaction (Figure 4A).

Next, we performed confocal microscopy to assess whether CoA supplementation alters the subcellular localization patterns of endogenous TXNRD2 and TXN2 (Figure 4B). The TXNRD2 and TXN2 proteins individually localized to mitochondria, as indicated by the COX IV mitochondrial marker. Upon CoA supplementation, we observed increased spatial overlap between TXNRD2 and TXN2 signals within mitochondria, consistent with enhanced interaction (Figure 4B). In contrast to the CoAlation of TXNRD2, other ferroptosis regulators such as FSP1 and GPX4 exhibited no detectable CoAlation (Supplemental Figure 4B).

To verify whether TXNRD2 can be CoAlated in vitro, we purified V5-tagged TXNRD2 proteins from HT-1080 cells overexpressing TXNRD2-V5 and incubated them with oxidized CoA using a previously published protocol (6). The purified proteins were then separated using native gel and blotted with anti-CoA antibody. We confirmed that TXNRD2, indeed, was robustly CoAlated, because treatment with oxidized CoA led to a marked shift in migration behaviors, suggesting structural changes (Supplemental Figure 4C). Next, we ran oxidized CoA-treated TXNRD2 in nonreducing gel and stained with anti-CoA antibody. Consistently, we found that treatment with oxidized CoA dramatically increased TXNRD2 CoAlation. This effect was abolished by DTT, which disrupted the disulfide bond of CoAlation (Figure 4C). Then we examined whether CoAlation of TXNRD2 affects its enzymatic activity (Figure 4D). Indeed, using the in vitro TXNRD assay, we found that CoAlation on TXNRD2 protein increased enzymatic activity. This effect could be completely inhibited by the TXNRD inhibitor auranofin (35) (Figure 4D), confirming the specificity. These results suggest CoA supplement can increase CoAlation on TXNRD2 and TXNRD2-TXN2 interaction.

### CoAlation on Cys-483 of TXNRD2 determines its enzymatic activity.

**Figure 5:**
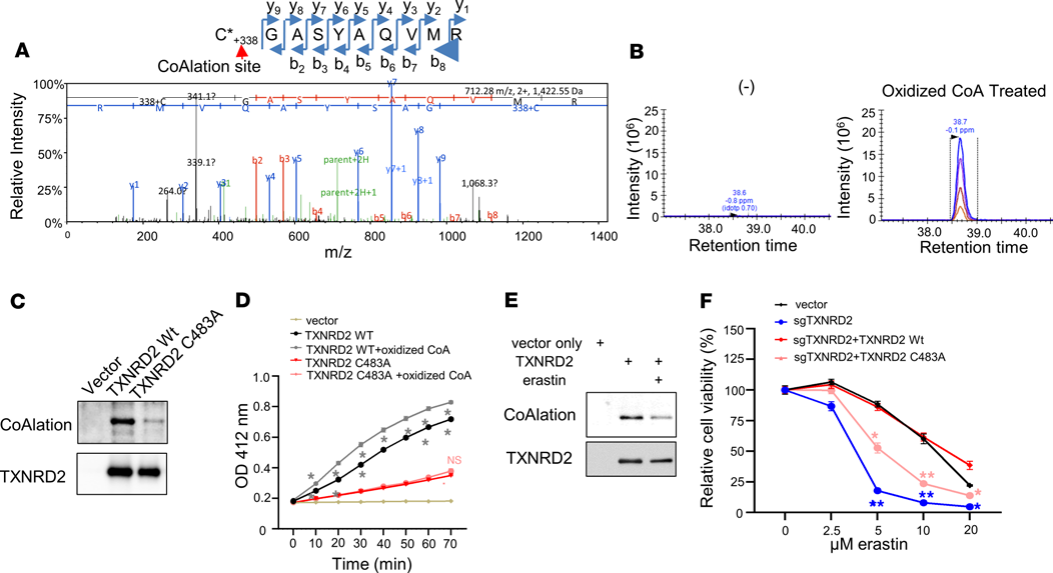
CoAlation of Cys-483 on TXNRD2 protein regulates TXNRD activity. (**A**) Tandem MS identified the CoA-modified peptide C*GASYAQVMR, localizing the modification to the N-terminal cysteine. (**B**) Relative quantification confirmed increased abundance of the modified peptide in oxidized CoA-treated versus DMSO samples, confirming C-483 CoAlation. (**C**) Mutation of Cys-483 to alanine (C483A) abolished most TXNRD2 CoAlation. Wild-type and mutant proteins were analyzed via nonreducing PAGE and Western blot. (**D**) The C483A mutation also abolished TXNRD activity, which could not be rescued by oxidized CoA. (**E**) Erastin treatment (2 μM, 16 hours) reduced CoAlation levels in HT-1080 cells overexpressing TXNRD2. (**F**) Cys-483 is essential for TXNRD2’s anti-ferroptotic function. Cells with TXNRD2 knockout were reconstituted with wild-type or C483A mutant, then treated with erastin (20 hours); only wild-type restored viability. (**D** and **F**) Data were analyzed by 2-way ANOVA and Šídák’s test; *n* = 3 biological replicates. *P < 0.05, **P < 0.01. Data presented as mean ± SEM. *m*/*z*, mass/charge ratio.

To identify the particular CoAlated residue(s) on TXNRD2 protein, we performed a bottom-up MS experiment on oxidized, CoA-treated, tryptic-digested TXNRD2 protein (Figure 5, A and B, and Supplemental Figure 5A). Compared with the untreated control, we identified a dynamic mass modification of +338.07 Da corresponding to fragmented CoA on Cys-483 of TXNRD2 (Figure 5, A and B, and Supplemental Figure 5A). Although the searches also included full-length CoA (+762.08 Da), full-length CoA was not identified in these data, possibly due to the instability of the intact structure in the gas phase, causing in-source fragmentation between phosphate groups.

The 3D structure of TXNRD2 has been solved (36), and Cys-483 is highly conserved during evolution and does not participate in forming intramolecular disulfide bonds (36). Cys-483 has also been postulated to have a potential redox regulatory role (36). To identify the function of this CoAlation residue, we used site-directed mutagenesis to replace Cys-483 with alanine (C483A) on TXNRD2 and transduced wild-type and the C483A mutant into HT-1080 cells (Figure 5C). Indeed, after protein purification, we found that wild-type TXNRD2 protein had robust endogenous levels of CoAlation signal, which was mostly abolished in C483A mutant (Figure 5C). Similarly, the protein-protein interaction between TXNRD2 and TXN2 was largely decreased by C483A (Supplemental Figure 5B). Also, this endogenous CoAlation signal can be removed by β-mercaptoethanol treatment, thereby disassociating CoA from modified Cys-483 and further demonstrating the specificity on this CoAlation signal (Supplemental Figure 5C). Therefore, we tested whether CoAlation on Cys-483 determines the enzymatic activity of TXNRD2 (Figure 5D). Compared with the wild-type TXNRD2 protein, the TXNRD activity of the C483A CoAlation-null mutant was significantly decreased (Figure 5D). Importantly, this point mutation (C483A) largely abolished the increased TXNRD activity associated with the treatment of oxidized CoA seen in wild-type TXNRD2 (Figure 5D). Collectively, these data suggest CoAlation on Cys-483 of TXNRD2 protein determines its TXNRD activity.

Next, we tested whether CoAlation on TXNRD2 protein could be modulated by cystine/cysteine availability. In TXNRD2-overexpressing HT-1080 cells treated with erastin or cystine deprivation, we found that these treatments, indeed, decreased CoAlation on purified TXNRD2 (Figure 5E and Supplemental Figure 5D). Similarly, inhibition of CoA biosynthesis by PANKi reduced the CoAlation of TXNRD2 protein (Supplemental Figure 5E).

To demonstrate that CoAlation on TXNRD2 determines cell viability, we performed CRISPR to knock out endogenous TXNRD2 (Supplemental Figure 5F). By treating these cells with erastin or a combination of BSO and PANKi, we found that knockout of TXNRD2 sensitized cells to ferroptosis, as determined by cell viability (Figure 5F and Supplemental Figure 5G) or mitochondrial lipid peroxidation (Supplemental Figure 5H). Next, the CRISPR-resistant wild-type or C483A mutant TXNRD2 was re-expressed at similar levels in the TXNRD2-null cells (Supplemental Figure 5F). The erastin or BSO/PANKi sensitizing effects in the TXNRD2-null cells were fully rescued by wild-type TXNRD2 but not C483A mutant in terms of viability (Figure 5F and Supplemental Figure 5G) and mitochondrial lipid peroxidation (Supplemental Figure 5H). These data suggest CoAlation on the Cys-483 of TXNRD2 protein regulates its enzymatic activity and ferroptosis sensitivity.

To investigate how CoAlation increases TXNRD2 activity, we performed covalent docking on a homology model of human TXNRD2. The best-scoring docking pose showed a covalent bond between the thiol group of Cys-483 and the sulfur atom of CoA with a docking score of –4.531 kcal/mol. In addition to Cys-483, CoA also interacts with several residues at the dimerization interface, including Glu435, Glu472, Cys500, and Ser501, in the 2D interaction diagram (Supplemental Figure 5I). These interactions suggest CoA binding could influence the conformational flexibility of the enzyme near its active site.

To assess the stability and dynamic behavior of the TXNRD2–CoA complex, we performed a 250 ns molecular dynamic simulation. The complex reached equilibrium within the first 50 ns of molecular dynamic simulations and remained stable throughout the rest of the simulation. Importantly, the covalent bond between CoA and Cys-483 was maintained for the entire duration of the simulation, supporting the chemical feasibility of this interaction under physiological conditions. Notably, CoA formed a network of stabilizing noncovalent interactions with the surrounding residues. The primary amine on the adenine of CoA engaged in hydrogen bonding with Gly476. The pantothenate –OH group engaged in hydrogen bonding with Arg492, and its adjacent carbonyl oxygen engaged in hydrogen bonding with Gln489. Additionally, the phosphate group at the 3′ position of the ribose ring of CoA established salt bridges with Lys482. Also, the adenine ring makes a π-π stacking interaction with His497 (Supplemental Figure 5J). Together, these interactions contribute to the robust structural integrity of the TXNRD2-CoA complex.

Superimposition of the initial and final structures (Supplemental Figure 5K) revealed a notable conformational change in the C-terminal region, specifically in residues Glu412 to Thr518. This region includes key elements involved in catalytic activity and substrate recognition (36). The conformational stabilization observed upon CoA binding suggests a potential mechanistic explanation for the enhanced enzymatic activity. CoA may restrict local flexibility or promote a catalytically favorable alignment of the C-terminal tail, thereby increasing TXNRD2 efficiency.

### Disruption of CoA metabolism by PANK inhibition leads to mitochondrial lipid peroxidation and ferroptosis.

**Figure 6:**
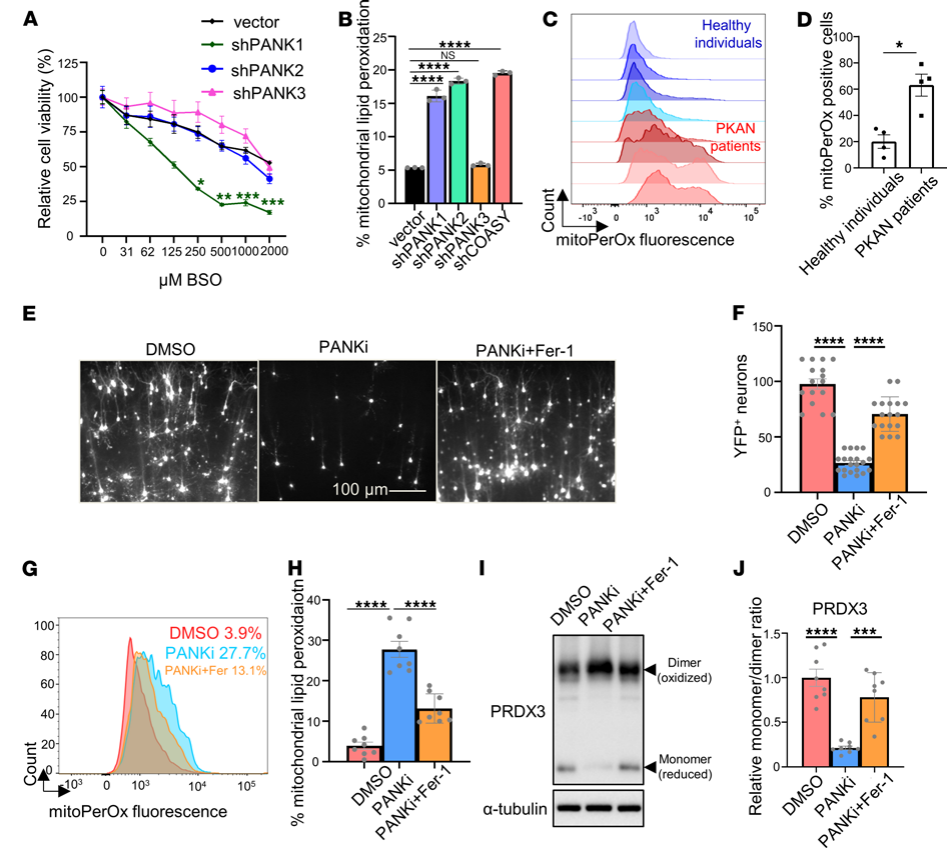
Disruption of CoA biosynthesis in PKAN fibroblasts and OBSC leads to mitochondrial lipid peroxidation. (**A**) *PANK1* knockdown sensitized HT-1080 cells to BSO treatment. HT-1080 cells with shRNA targeting *PANK1*, *PANK2*, or *PANK3* were treated with BSO for 3 days for Cell-Titer Glo assay. Data were analyzed by 2-way ANOVA and Šídák’s multiple comparisons; *n* = 3 independent biological replicates. (**B**) *PANK1*, *PANK2*, and *COASY* knockdown in HT-1080 cells showed an increase in mitochondrial lipid peroxidation. (**C** and **D**) Primary fibroblasts from patients with PKAN and age- and sex-matched unaffected individuals were stained with mitoPerOx, a probe that detects mitochondrial lipid peroxides. Representative images of mitoPerOx staining in (**C**) demonstrate a visibly higher signal intensity in PKAN fibroblasts. Quantification of the percentage of mitoPerOx^+^ cells is presented in (**D**), confirming a significant increase in mitochondrial lipid peroxidation in fibroblasts from patients with PKAN. Data were analyzed by unpaired *t* test. (**E** and **F**) The cell death triggered by inhibiting CoA using PANKi in OBSC was rescued by Fer-1. OBSCs transfected with YFP were treated with PANKi (2.5 μM) or in combination with Fer-1 (2 μM, 24 hours). Neuronal viability was assessed by fluorescence microscopy: the number of YFP^+^ neurons remaining after treatment was quantified by fluorescence microscopy as a measure of cell survival (**E** and **F**). Representative images of YFP^+^ neurons are shown in (**E**), and quantification is presented in (**F**). (**G** and **H**) The elevated mitochondrial lipid peroxidation by PANKi was rescued by Fer-1. After 1 day of treatment with PANKi (2.5 μM) and Fer-1 (2 μM), OBSC was stained with mitoPerOx (**G**) for quantification (**H**). (**I** and **J**) PANKi treatment of brain tissue sections repressed the levels of reduced form of PRDX3, which was rescued by Fer-1. OBSC treated with PANKi (2.5 μM) or in combination with Fer-1 (2 μM) were blotted with PRDX3 (**I**) and quantification (**J**). (**B**, **F**, **H**, and **J**) Data were analyzed by 1-way ANOVA and Tukey’s multiple comparisons. *P < 0.05, **P < 0.01, ***P < 0.001, and ****P < 0.0001. Data are mean ± SEM.

Mutations in human genes *PANK2* or *COASY*, 2 genes that encode proteins involved in the CoA biosynthesis pathway, lead to NBIA (2). In particular, mutations in *PANK2* lead to PKAN (2). The mammalian genome encodes 4 isozymes that possess pantothenate kinase activity (PANK1α, PANK1β, PANK2, and PANK3). Their tissue expression patterns and subcellular localization have been extensively documented (3). However, the respective cellular roles of each PANK protein and the unique cellular role of *PANK2* mutations in tissue-specific vulnerability leading to PKAN are still unclear. By selectively targeting the GSH synthesis using BSO, we found that suppressing the mitochondrial TXN system by inhibiting PANK or knocking down COASY significantly increased ferroptosis (Figure 3, C and D) and mitochondrial lipid peroxidation (Figure 3, E–G). To investigate the role of individual PANKs in CoA-directed TXN regulation, we knocked down individual PANK proteins in HT-1080 cells and found that only PANK1 knockdown increased BSO-induced ferroptosis (Figure 6A and Supplemental Figure 6A) without affecting cell proliferation (Supplemental Figure 6B). Surprisingly, by using BSO to selectively target the GSH system, the cell viability of fibroblasts from patients with PKAN substantial decreased, whereas the fibroblasts from healthy individuals were unaffected (Supplemental Figure 6C). Importantly, CoA supplementation rescued the BSO-induced cell death in fibroblasts from patients with PKAN (Supplemental Figure 6D).

Next, in HT-1080 cells, the knockdown of PANK1 and PANK2, but not PANK3, increased mitochondrial lipid peroxidation to the same level as COASY knockdown (Figure 6B), which was abolished by CoA supplement (Supplemental Figure 6E). We also compared mitochondrial ROS and mitochondrial lipid peroxidation in fibroblasts between patients with PKAN and healthy individuals (Figure 6, C and D, and Supplemental Figure 6, F and G). Surprisingly, we observed increased mitochondrial lipid ROS and mitochondrial lipid peroxidation in fibroblasts from patients with PKAN (Figure 6, C and D, and Supplemental Figure 6, F and G). Although *PANK2* mutation/knockdown may exhibit different responses in cell viability to the stress of BSO-mediated GSH depletion, these data suggest a consistent increase in mitochondrial lipid peroxidation upon *PANK2* mutation/knockdown in PKAN fibroblast and HT-1080 cells.

To bridge the gap between cell-based assays and whole-animal models, OBSC has been developed to investigate the mechanisms of diseases and accelerate drug development (37–39). OBSC is an ex vivo model system that preserves the 3D cytoarchitecture and cell-cell interactions of brain tissue. Prepared from neonatal or juvenile rat brains, the sections are typically 100–400 μm thick and so retain a diverse cellular composition, including neurons, astrocytes, microglia, and endothelial cells, enabling the study of complex brain processes in a physiologically relevant context (37–39). OBSC also serves as a model system for studying ferroptosis effects in neurons (40). To mimic the human CoA metabolic defect, we treated rat OBSC with PANKi to inhibit CoA biosynthesis. By monitoring the number and viability of neurons after transfecting OBSC with yellow fluorescent protein (YFP), we observed that the number of neurons was significantly reduced after PANKi treatment, which could be rescued by the ferroptosis inhibitor, Fer-1 (Figure 6, E and F). These data suggest that inhibition of the TXN system by PANKi itself is sufficient to trigger ferroptosis in the OBSC model. Subsequently, we measured mitochondrial lipid peroxidation and found it was consistently increased upon PANKi treatment, which could be abolished through the concurrent application of Fer-1 (Figure 6, G and H). These data suggest the PANKi triggered mitochondrial lipid peroxidation in neurons in the OBSC model. Finally, we measured the redox status of the mitochondrial PRDX3 and found that PANKi downregulated the levels of reduced monomers, which were restored by Fer-1 treatment (Figure 6, I and J).

PANKi treatment itself did not induce ferroptosis in the 6 cancer cell lines (Supplemental Figure 6H); however, our data suggest inhibiting CoA biosynthesis may trigger ferroptosis by inducing mitochondrial lipid peroxidation in rat neurons in the OBSC model. Consistently, we found that CoA inhibition also triggered cell death in an N27 rat dopaminergic neural cell line (Supplemental Figure 6I), suggesting that neuronal cells are more sensitive to CoA inhibition than are cancer cells.

Next, we compared the expression of the mitochondrial TXN system and important ferroptosis markers in patients with PKAN. PKAN, a rare genetic disorder, occurs in 1 to 3 out of 1 million individuals (3); we were able to acquire RNA and protein samples from the fibroblasts of 7 patients with PKAN in comparison with healthy unaffected individuals. Although we did not observe significant differences at RNA levels for *TXNRD2*, *TXN2*, *SLC7A11*, *GCLC*, *GPX4*, and *PTGS2* (Supplemental Figure 6J), we found that TXNRD2 and GPX4 proteins were significantly lower in patients with PKAN (Supplemental Figure 6, K and L), highlighting a potential clinical relevance of linking CoA synthesis with the mitochondrial TXN system and GSH system.

## Discussion

Since ferroptosis was first described as an iron-dependent, oxidative form of regulated cell death triggered by erastin and other small molecules (41), the role of cystine import and GSH synthesis in suppressing lipid peroxidation through GPX4 has been extensively characterized. Subsequent studies have identified GPX4-independent ferroptosis defense mechanisms, including FSP1-mediated ubiquinone regeneration (9, 42) and the dihydroorotate dehydrogenase pathway (12). These studies highlight the existence of parallel, compartmentalized systems that restrict lipid peroxidation in different organelles. Our study adds to this framework by identifying the xCT-cysteine/CoA/TXNRD2 axis as a distinct mitochondrial lipid peroxidation defense mechanism, emphasizing the role of CoA in maintaining mitochondrial redox homeostasis.

CoA was first identified as a ferroptosis inhibitor in p53-mutant cells with increased CoA levels that protect against erastin- and glutamate-induced ferroptosis (15). In pancreatic tumor cells, CoA was proposed to protect ferroptosis by enhancing the production of CoQ10, (8). In this study, we found that CoA serves as a protective agent against ferroptosis induced by class I FINs, but not other classes of FINs, in multiple cancer cell lines (Figure 1C and Supplemental Figure 1, A–F). Previous studies have found that class I FINs require mitochondria and their lipid peroxidation to trigger ferroptosis via an unknown mechanism (11, 12). Curiously, although BSO efficiently reduces GSH synthesis, BSO is a considerably less potent FIN to trigger ferroptosis. One group proposed that GSH inhibition by BSO led to the accumulation of intracellular cysteine levels, which were used by the TXN system to suppress ferroptosis (14). However, the mechanism by which cysteine regulates the TXN system has not been fully elucidated (14). Instead of direct fueling of TXN system by cysteine, our findings suggest cysteine is incorporated for the biosynthesis of CoA, which protects cells from ferroptosis by CoAlation of TXNRD2 to maintain a mitochondrial TXN function and prevent mitochondrial lipid peroxidation. Thus, xCT-cysteine/CoA/TXNRD2 is an important independent branch of the ferroptosis defense mechanism, distinct from the xCT/GSH/GPX4 axis that neutralizes mitochondrial lipid peroxidation.

Interestingly, we observed a striking difference in the effects of PANK inhibition between neurons (Figure 6E and Supplemental Figure 6I) and cancer cell lines (Supplemental Figure 6H). Although inhibition of either xCT/GSH/GPX4 (by BSO) or xCT-cysteine/CoA/TXNRD2 (by PANKi) alone can be tolerated in cancer cells, their combination leads to synthetic lethality (Figure 3D). Cancer cells were typically isolated and selected for a strong xCT/GSH/GPX4 axis and robust GSH synthesis that buffers ROS accumulation (43). In contrast, neurons have a weaker xCT/GSH/GPX4 axis due to limited cystine uptake and lower expression of GCL, and rely on astrocytes to maintain GSH levels (44). Thus, PANKi-induced disruption of CoA biosynthesis in neurons may synergize with their poor GSH metabolism to drive cell death, while cancer cells remain viable due to their compensatory GSH systems.

Our data indicate CoA protects against ferroptosis induced by class I FINs (e.g., erastin, sulfasalazine) but not class II or III FINs, such as GPX4 inhibitors (RSL3, ML162, or FIN56). These findings align with previous studies demonstrating that mitochondria play an essential role in cysteine-deprivation-induced ferroptosis but are dispensable for ferroptosis induced by GPX4 inhibition (11). Mechanistically, class I FINs rely on the mitochondrial TCA cycle and electron-transfer chain activity to drive lipid peroxide generation, whereas class II FINs bypass mitochondrial function and directly inhibit GPX4, the terminal component of the xCT/GSH/GPX4 axis. Because CoA regulates the mitochondrial TXN system to suppress mitochondrial lipid peroxidation, its effect is limited to the ferroptosis mechanism involving mitochondria.

Protein CoAlation was first identified in 2017 as a reversible PTM in eukaryotic and prokaryotic cells in response to oxidizing environments and metabolic stress (6, 45). So far, CoAlation has been found to inhibit the activity of modified proteins implicated in metabolic or signaling pathways (7). The inhibitory effects of CoAlation have been demonstrated in aconitase, creatine kinase, pyruvate dehydrogenase kinase 2, GADPH, hydroxymethylglutaryl-CoA synthase, Aurora kinase A, and metastasis suppressor protein NME1 (6, 7, 46–48). In contrast to these reported inhibitory effects, we found that CoAlation on Cys-483 of TXNRD2 increased its TXNRD activity, which may involve an allosteric mode of activation (Figure 5D). Importantly, CoAlation levels on TXNRD2 respond to the availability of cysteine and CoA as a nutrient-sensing mechanism (Figure 5E and Supplemental Figure 5, D and E). Upon the erastin-induced ferroptosis, CoAlation on TXNRD2 determined cell viability (Figure 5F). Importantly, Cys-483 is highly conserved among mammalian TXNRDs (36) and is surface exposed. Therefore, this residue has been postulated to have a potential redox regulatory role (36). Our findings confirm this prediction that CoAlation on Cys-483 is a nutrient-sensing mechanism that adjusts the enzymatic activity of TXNRD2 and ferroptosis sensitivity based on the levels of CoA. Given that Cys-483 is highly conserved during evolution (36), redox status regulation by CoAlation may have broader impacts on other TXNRDs across different species, because CoA biosynthesis is an ancient pathway conserved across different domains of life (49).

These mechanistic insights into CoA/CoAlation/TXNRD2 signaling offer a promising direction for therapeutic intervention. Small molecules that modulate CoA synthesis or mimic CoAlation on TXNRD2 could potentially be developed as ferroptosis inhibitors specifically targeting the redox status of mitochondria, especially for neurodegenerative diseases in which ferroptosis plays a pathogenic role (50). Conversely, disrupting this pathway may sensitize cancer cells to ferroptosis-inducing agents, offering a synthetic lethality approach when combined with xCT inhibitors (9, 51). Furthermore, because CoAlation responds to cellular metabolic state (6), it may serve as a pharmacodynamic marker to stratify patients or monitor treatment efficacy in ferroptosis-targeted therapies, much like redox-sensitive PTMs that are increasingly used to evaluate drug response in metabolic and oxidative stress conditions (52).

NBIA is a group of rare neurodegenerative disorders with increased basal ganglia iron on brain magnetic resonance imaging (53). NBIA is associated with mutations in different enzymes involved in the CoA biosynthesis, including *PANK2* and *COASY*. Mutations in *PANK2*, a key mitochondrial enzyme involved in CoA biosynthesis, account for approximately half of NBIA cases (53). Given that iron is essential for ferroptosis via the Fenton reaction, it is reasonable to speculate on a relationship between ferroptosis and PKAN/NBIA. Consistently, PKAN astrocytes were found to be prone to ferroptosis with higher levels of oxidized proteins and malondialdehyde (54). However, a direct connection between disrupted CoA metabolism and ferroptosis during PKAN is still lacking. In this study, we found that *PANK2* knockdown increased mitochondrial lipid peroxidation in HT-1080 cells (Figure 6B). Even though patients with PKAN showed no reduction in CoA levels in fibroblasts (3), we still observed increased mitochondrial lipid peroxidation (Figure 6, C and D) that may predispose these cells to ferroptosis. Indeed, fibroblasts from patients with PKAN are more sensitive to BSO-mediated GSH depletion (Supplemental Figure 6C). We also found that OBSC treated with PANKi exhibited many features associated with PKAN/NBIA, including a reduced number of neurons, increased mitochondrial lipid peroxidation, and disrupted TXN systems (Figure 6, E–J). Importantly, the application of Fer-1 to rescue these phenotypes implies that PANKi-induced cell death is caused by ferroptosis (Figure 6, E–J). These data also suggest that we have developed an OBSC model targeting all PANKs to complement current models of PKAN and NBIA. With the development of PANK2-specific inhibitors in the future or genetic removal of *PANK2*, this OBSC model can be further developed to reveal the mechanistic underpinnings and therapeutic agents.

These findings not only support ferroptosis as a potential pathogenic mechanism in NBIA/PKAN, but also open the door to testing various ferroptosis inhibitors, like Fer-1, as candidate neuroprotective agents in these disorders. Moreover, the pharmacologic strategies to modulate CoAlation, such as developing novel PANKis or small-molecule CoA analogs, could offer an innovative means to overcome ferroptosis resistance in a disease- or tissue-specific manner. Together, inhibiting ferroptosis and targeting CoAlation pathways may have significant therapeutic implications.

## Methods

### Sex as a biological variable.

Our study examined sectioned brain tissue of both male and female rats, and similar findings are reported for both sexes.

### Mass spectrometry.

CoA and acetyl-CoA were extracted from 30 million cells per sample using previously published methods (55). The cell pellets were treated with 300 μL of ice-cold 0.1 M KH_2_PO_4_. Methanol containing 10% acetic acid was added to the cell pellets, followed by solid phase extraction. For the solid phase extraction process, a 1 mL ion exchange cartridge packed with 100 mg of 2–2(pyridyl)ethyl silica gel (Sigma) was activated with 1 mL of methanol and equilibrated with 1 mL of buffer A (methanol/H_2_O 1:1, containing 5% acetic acid). The sample extract was loaded onto the cartridge, and additional steps with buffer A, buffer B (methanol/H_2_O 1:1, 50 mM ammonium formate), buffer C (methanol/H_2_O 3:1, 50 mM ammonium formate), and methanol were performed. The eluent (3 mL) was collected and dried using N_2_ gas. The resulting dried residue was resuspended in 100 μL of H_2_O for liquid chromatography–tandem MS (LC-MS/MS) analysis (55, 56).

For CoAlation site identification on TXNRD2, HT-1080 cells overexpressing TXNRD2-v5 were lysed with NP-40 buffer containing 25 mM *N*-ethylmaleimide (NEM; 23030, Thermo Fisher Scientific) and protease inhibitor (04693116001, Roche) at 4°C with constant shaking. The lysates were then centrifuged at 21,000*g* for 10 minutes, and the supernatant was purified by the V5-tagged Protein Purification Kit (3317, Medical & Biological Laboratories [MBL]) and eluted by v5 peptide. Purified TXNRD2 was incubated with 10 mM oxidized CoA in 50 mM Tris-HCl (pH 7.5) at room temperature for 30 minutes. The samples with or without oxidized CoA treatment were then resolved on a 12% nonreducing SDS-PAGE gel. After colloidal blue staining (LC6025, Thermo Fisher Scientific), the bands at approximately 50 kDa were cut for MS.

The bands obtained from SDS-PAGE gels underwent in-gel tryptic digestion, following established protocols. After digestion, peptides were dehydrated and then reconstituted in a solution containing 0.2% formic acid and 2% acetonitrile at a volume of 12 μL. Each resulting sample underwent chromatographic separation using Waters MClass ultra performance liquid chromatography, using a 1.7 μm HSS T3 C18 75 μm I.D. × 250 mm reversed-phase column (operated in nano-flow mode). The mobile phase for chromatography consisted of 2 components: (a) 0.1% formic acid in water and (b) 0.1% formic acid in acetonitrile. Injection of 3 μL of the sample commenced the process, with peptides initially captured for 3 minutes on a 5 μm Symmetry C18 180 μm I.D. × 20 mm column at a rate of 5 μL/min, with a composition of 99.9% A. Following this, an analytical column was engaged, and a linear elution gradient ranging from 5% B to 40% B occurred over 60 minutes at a flow rate of 400 nL/min.

The Fusion Lumos mass spectrometer (Thermo Fisher Scientific) was connected to the analytical column via an electrospray interface, functioning in a data-dependent acquisition mode. The instrument settings included a precursor MS scan from a mass/charge ratio of 375–1,500 at *R* = 120,000 (target automatic gain control [AGC] = 2 × 10^5^; maximum injection time [IT] = 50 ms), with subsequent MS/MS spectra acquired in the ion trap (target AGC = 1 × 10^4^, maximum IT = 100 ms). Higher-energy C-trap dissociation energy settings were consistently maintained at 30 V for all experiments, and a dynamic exclusion of 20 seconds was implemented to avoid reanalysis of previously fragmented precursor ions.

The LC-MS/MS data files obtained were processed using Proteome Discoverer 3.0 (Thermo Fisher Scientific) and subsequently subjected to independent Sequest database searches against a comprehensive human protein database. This database included both forward (*n* = 20,260 entries) and reverse entries for each protein. The search parameters allowed for 2 ppm tolerance for precursor ions and 0.8 Da tolerance for product ions, using trypsin specificity with allowance for up to 2 missed cleavages. Dynamic mass modifications were defined for cleaved CoA (+338.07 Da on C) and full-length CoA (+762.08 Da on C). All spectra derived from the searches were imported into Scaffold (version 5.3; Proteome Software), where scoring thresholds were adjusted to maintain a peptide false discovery rate of 1%, using the PeptideProphet algorithm. Additionally, the raw data were imported into Skyline (MacCoss Lab, University of Washington) for the purpose of measuring extracted ion chromatograms of modified peptides.

### Chemicals.

The following chemicals were used in this work: Auranofin (A6733, MilliporeSigma); ferroptocide (F1293, Tokyo Chemical Industry); CoA (F15115, Astatech); erastin (5499, Bio-techne); BSO (B2515, MilliporeSigma); PANKi (31002), RSL3 (19288), ML162 (20455), FIN56 (25180), Fer-1 (17729,), lipro (17730), and iFSP1 (29483) — all from Cayman Chemical; Trolox (218940010, Thermo Fisher Scientific); lovastatin (S2061), simvastatin (S1792), brequinar (S3565), teriflunomide (S4169), methotrexate (S1210), Compound C (S7306), TOFA (S6690), and etomoxir sodium salt (S8244) — all from Selleckchem; and 3-deazaadenosine (HY-W013332A); 3-deazaneplanocin A (HY-12186), and adenosine dialdehyde (HY-123055) — all from Medchemexpress.

### In vitro assay.

To determine TXN activity in cell lysate, after the treatment of erastin, CoA, or in combination, 30 million cells per sample were washed with PBS and homogenized in 500 μL of cold buffer (100 mM Tris-HCl, pH7.5, 1 mM EDTA) with protease inhibitor. After 15 minutes of centrifugation (10,000*g*, 4°C), the TXN activity in the supernatant was determined by the Thioredoxin Fluorometric Activity Assay Kit (500228, Cayman Chemical). To determine TXNRD activity in cell lysate, after the treatment of erastin, CoA, or in combination, 30 million cells per sample were washed with PBS and homogenized in 500 μL of cold buffer (50 mM potassium phosphate, pH 7.4, 1 mM EDTA) with protease inhibitor. After 15 minutes of centrifugation (10,000*g*, 4°C), the TXN activity in the supernatant was determined by Thioredoxin Reductase Colorimetric Assay Kit (10007892, Cayman Chemical). To determine TXNRD activity in purified TXNRD2 protein, HT-1080 cells transduced with TXNRD2 wild-type or C483A mutant were lysed with NP-40 buffer with protease inhibitor and 25 mM NEM. After centrifugation, the supernatant was purified by the V5-tagged Purification Kit, version 2 (3317, MBL). The purified TXNRD2 protein was resolved by nonreducing SDS-PAGE to confirm equal amount of input for the Thioredoxin Reductase Colorimetric Assay Kit.

### Cell culture.

HT-1080, HEK-293, 786-O, H1975, RCC4, and MDA-MB-231 cells were obtained from Duke University’s Cell Culture Facility and authenticated by short tandem repeat profiling. Prior to freezing, these cell lines were authenticated using short tandem repeat DNA profiling to ensure their identity, and they were confirmed to be free from mycoplasma contamination by the Cell Culture Facility. The cells were cultured for less than 6 months. Human fibroblasts from healthy individuals were obtained from Coriell Institute for Medical Research. All fibroblast collection was part of Duke’s IRB-approved protocol (no. Pro00101047). All cell lines were maintained in a humidified incubator at 37°C with 5% CO_2_ in DMEM (GIBCO-11995, Thermo Fisher Scientific) supplemented with 10% heat-inactivated FBS (10082147, Thermo Fisher Scientific) and antibiotics (streptomycin, 10,000 IU/mL and penicillin, 10,000 IU/mL; 15140122, Thermo Fisher Scientific).

### Constructs and lentivirus viral infections.

siRNAs targeting human *COASY*, *TXN1*, *TXN2*, *ACSL3*, and *ACSL4* RNA were obtained from Dharmacon (catalog D-006751-01, D-006751-02, M-006340-01-0005, M-017448-00-0005, M-010061-00-0005, and M-009364-00-0005). The guide RNA targeting TXNRD2, TXN2, and GCLC was purchased from MilliporeSigma (catalog HSPD0000063630, HSPD0000075931, and HSPD0000016313 in GeCKOv2 all-in-1 lentiviral plasmid). The TXNRD2 cDNA in PLX304 was procured from the DNASU Plasmid Repository (catalog HsCD00446697). To generate guide RNA-resistant TXNRD2 (with synonymous mutation) and point mutant (TXNRD2 C483A), we used the QuikChange II XL Site-Directed Mutagenesis Kit (200521, Agilent Technologies). Lentivirus expressing specific constructs was produced by transfecting HEK-293 cells in 6-well plates with a 1:1:0.1 ratio of lentiviral vector/pMD2.G/psPAX2 using the TransIT-LT1 transfection reagent (MiRus). Subsequently, the lentivirus was filtered through a cellulose acetate membrane (0.45 μm; 28145-481, VWR), and 250 μL of medium containing the lentivirus was added to a 60 mm dish of the indicated cells along with polybrene (8 μg/mL) and selected with puromycin or blasticidin.

### Cell viability and cytotoxicity.

Cell viability was assessed using the CellTiter-Glo luminescent cell viability assay (Promega) according to the manufacturer’s instructions. Briefly, 15 μL of CellTiter-Glo substrate was added to cells cultured in a 96-well plate with 100 μL of medium, followed by 10 minutes of shaking. The signal intensity was measured using a chemiluminescence plate reader. For quantification of cell death, the CellTox Green assay (Promega) was performed. The dye was added to the medium at a 1:1,000 dilution, and cell death was quantified using a fluorescence plate reader. To determine the level of GSH, the GSH/GSSG-Glo Assay (Promega) was conducted according to the manufacturer’s guidelines. Briefly, the samples in a 96-well plate were treated with a lysis reagent specific to each measurement set: total GSH lysis reagent for total GSH measurement and oxidized GSH lysis reagent for glutathione disulfide (GSSG) measurement. Afterward, luciferin-generation reagent was added to all wells, and the assays were mixed and incubated for 30 minutes. Following this, a luciferin detection reagent was added to all wells, and luminescence was measured after a 15-minute incubation. GSH/GSSG ratios were directly calculated from the luminescence measurements (in RLUs).

### Western blots.

Protein concentrations were quantified by bicinchoninic acid assay (23227, Thermo Fisher Scientific). After protein extraction, the samples were loaded on 12% native, nonreducing, or reducing SDS-PAGE gels, transferred to a PDVF membrane, blocked with 5% nonfat milk in 1× TBST, and incubated with primary antibodies overnight at 4°C. The primary antibodies and their dilutions were COASY (1:1,000; sc-393812, Santa Cruz Biotechnology); PRDX1 (1:1,000; 15816-1-AP, Proteintech); PRDX3 (1:1,000; 10664-1-AP, Thermo Fisher Scientific); GAPDH (1:2,000; sc-25778, Santa Cruz Biotechnology); TXNRD1 (1:1,000; 11117-1-AP, Santa Cruz Biotechnology); TXNRD2 (1:1,000; PA529458, Thermo Fisher Scientific); v5 (1:1,000; MA5-15253, Thermo Fisher Scientific); and α-tubulin (1:1,000; sc-32293, Santa Cruz Biotechnology). We generated the CoAlation antibody used in this study, which is commercially available (clone 567A19-12G9, PT-mAb-CoA; ProtTech). TXNRD2 protein was purified from HT-1080 cells overexpressing TXNRD2-v5 by the V5-tagged Protein Purification Kit, version 2 (3317, MBL). For TXNRD2 purification, HT-1080 cells were lysed by NP-40 buffer with 25 mM NEM (23030, Thermo Fisher Scientific) and protease inhibitor (04693116001, Roche) at 4°C with constant shaking. The lysates were then centrifuged at 21,000*g* for 10 minutes, and the supernatant was purified by the V5-tagged Protein Purification Kit. For PRDX blots, the cells were washed with PBS and incubated in NEM buffer (40 mM HEPES [pH 7.4], 50 mM NaCl, 1 mM EDTA, 1 mM EGTA, 100 mM NEM, protease inhibitor) for 10 minutes. CHAPS (1%) was then added to lyse the cells for quantification.

### Confocal microscopy.

IGROV-1 cells with or without CoA supplement were washed twice with PBS and fixed in 3.7 % paraformaldehyde for 20 minutes. The samples were permeabilized and blocked with 0.2% Triton X-100 and 2% BSA for 30 minutes. Primary antibodies were incubated with the cells for 1 hour. Confocal microscopy was performed using LSM 880 with Airyscan (Zeiss). The following antibodies were used: TXNRD2 (1:100; PA529458, Thermo Fisher Scientific); TXN2 (1:100; sc-133201, Santa Cruz Biotechnology); and COX IV with direct AF594 conjugate (1:100; 8692S, Cell Signaling Technology).

### Quantitative real-time PCR.

The RNA extraction and purification were carried out using the RNeasy Mini Kit (Qiagen), following the manufacturer’s recommended protocol. Subsequently, reverse transcription to cDNA was performed using random hexamers and SuperScript IV reverse transcriptase (Invitrogen). For quantitative real-time PCR analysis, the cDNA was combined with primers and Power SYBR Green PCR Mix (Thermo Fisher Scientific), and the reactions were run on a StepOnePlus Real-time PCR system (Thermo Fisher Scientific). Each sample was technically triplicated to obtain the mean ± SEM. The data presented are representative of a minimum of 2 independent experimental repetitions. Primer sequences are listed in Supplemental Table 1.

### Lipid peroxidation assay.

Lipid peroxidation was assessed using C11–boron-dipyrromethene (C11-BODIPY) staining following the manufacturer’s instructions (D3861, Thermo Fisher Scientific). Briefly, cells were exposed to either a vehicle control or treatments for 16 hours. Subsequently, the medium was replaced with 10 μM C11-BODIPY–containing medium and incubated for 1 hour. After harvesting, washing, and resuspension in PBS containing 1% BSA, the levels of lipid peroxidation were quantified using flow cytometry (FACSCanto TM II; BD Biosciences). Similarly, for mitochondrial lipid peroxidation, mitoPerOx (57) (200 nM; 18798, Cayman Chemical) containing medium was incubated with cells for 30 minutes.

### Protein structure preparation.

The sequence of *Homo sapiens* mitochondrial TXNRD2 was obtained from the NCBI database (NCBI ID: NP_006431.2). The Swiss-Model web server (https://swissmodel.expasy.org/) was used to model the dimeric structure based on the crystal structure of *Mus musculus* TXNRD (PDB_ID: 3DGZ; Protein Data Bank) as the template. In the monomeric model protein, the bond orders were adjusted, hydrogen atoms were added, charges were assigned, and the OPLS4 force field was used for optimization and energy minimization using Schrödinger Maestro.

### Molecular docking.

The LigPrep module in Schrödinger Maestro was used to generate the CoA structure. The 2D coordinates were converted to 3D, the force field geometry was optimized using the OPLS4 force field, and hydrogen atoms were added. Molecular docking of CoA on the monomeric TXNRD2 was performed using the Covalent docking module. The number of CoA docking poses was set to 10.

### Molecular dynamics simulations.

Molecular dynamics simulations were performed for the TXNRD2-CoA complex using the Desmond module of Schrödinger Maestro for 250 ns at 300 K and 1.01325 bar pressure. The complex was solvated in an orthorhombic box measuring 10 Å × 10 Å × 10 Å with the TIP3P water model, and the system was neutralized by adding Na^+^ ions.

### OBSCs.

The experiment involved the use of CD Sprague-Dawley rat pups at postnatal day 10, obtained from Charles River Laboratories. The rat brains were sliced into 250-μm coronal sections using vibratomes (Vibratome Co.) in chilled medium baths. Each rat brain yielded approximately 6 slices, which were further divided into hemicoronal slices. These slices were individually placed in multiwell plates, resulting in 12 brain slice assays per rat brain.

To establish the brain slice assays, the slices were explanted in an interface configuration using culture medium containing 15% heat-inactivated horse serum, 10 mM KCl, 10 mM HEPES, 100 U/mL penicillin/streptomycin, 1 mM MEM sodium pyruvate, and 1 mM l-glutamine in Neurobasal A (Invitrogen). Before use, the medium was filter sterilized at 0.22 μm.

The 12-well plates used for plating the slices provided culture support through a low concentration of agarose (0.5%; J.T. Baker). The slice cultures were maintained in humidified incubators at 32°C under 5% CO_2_.

To label healthy neurons, YFP was transfected using a biolistic device (Bio-Rad Helios Gene Gun). After the treatment of PANKi or a combination of PANKi with Fer-1, neurons expressing YFP were observed and visualized under fluorescence microscopy after 1 day of incubation. Neuronal viability was assessed by fluorescence microscopy; the number of YFP-positive neurons remaining after treatment was quantified as a measure of cell survival.

### Statistics.

The bar graphs display individual data points, each representing the number of biological replicates as indicated. The line graph includes the number of biological replicates specified in the figure legends. All data are presented as the mean ± SEM. Statistical significance was determined using GraphPad software with 1-way ANOVA and Tukey’s multiple-comparison test, 2-way ANOVA with Šídák’s multiple-comparison test, or a 2-tailed Student’s *t* test, as appropriate. Significance between samples is denoted in the figure legends.

### Study approval.

All animal procedures, including the preparation of OBSCs from Sprague-Dawley rat pups, were reviewed and approved by the Duke University IACUC, Durham, North Carolina, USA, under protocol number A233–23-11. Rats were housed in Duke’s Association for Assessment and Accreditation of Laboratory Animal Care–accredited animal care facility and received daily care under the supervision of veterinary staff from the Division of Laboratory Animal Resources. All animal experiments complied with the US Animal Welfare Act, the Public Health Service Policy on Humane Care and Use of Laboratory Animals, and the NIH *Guide for the Care and Use of Laboratory Animals* (National Academies Press, 2011).

Human primary dermal fibroblast lines from patients and healthy control individuals were obtained with written informed consent and collected under Oregon Health & Science University’s IRB-approved repository protocol 7232 (Oregon Health & Science University, Portland, Oregon, USA). These de-identified samples were shared with Duke University under appropriate material transfer agreements. Additional de-identified human fibroblast lines were obtained under an exempt protocol approved by the Duke University Health System IRB, Durham, North Carolina, USA (protocol Pro00101047) and are not considered human subjects research.

### Data availability.

All data and reagents supporting the study’s findings are available upon reasonable request. MS data are deposited in the ProteomeXchange Consortium (ID PXD062566). Values for all data points in graphs are reported in the Supporting Data Values file.

## Author contributions

CCL and JTC conceived the experiments and wrote the manuscript. CCL performed the majority of the experiments. JTC supervised the work. YTL, SYC, YS, YC, DED, TN, AAM, AB, LG, EJS, SRF, SYJ, VF, and GFZ collaborated in the discussion and experiments. CYL provided critical reagents. SJH and IG provided critical feedback.

## Supplementary Material

Supplemental data

Unedited blot and gel images

Supporting data values

## Version 1 07/22/2025

In-Press Preview

## Version 2 10/01/2025

Electronic publication
