# Supplementary material for: Coenzyme A protects against ferroptosis via CoAlation of mitochondrial thioredoxin reductase
Source: J Clin Invest. 2025 Jul 22;135(19):e190215. doi: 10.1172/JCI190215 (PMC12483566; doi:10.1172/JCI190215)
Supplement: Supplemental data [file jci-135-190215-s017.pdf]

Supplementary Materials for

Coenzyme A protects against ferroptosis via CoAlation of mitochondrial thioredoxin reductase

Chao-Chieh Lin[1,2], Yi-Tzu Lin[1,2], Ssu-Yu Chen[1,2], Yasaman Setayeshpour[1,2], Yubin Chen[1,2], Denise E. Dunn[3], Taylor Nguyen[3], Alexander A. Mestre[1,2,4], Adrija Banerjee[5], Lalitha Guruprasad[5], Erik J. Soderblom[6], Guo-Fang Zhang[7,8], Chen-Yong Lin[9], Valeriy Filonenko[10], Suh Young Jeong[11], Scott R. Floyd[3], Susan J. Hayflick[11,12], Ivan Gout[10,13], Jen-Tsan Chi[1, 2] .

Corresponding author: jentsan.chi@duke.edu

Lin_ Supplemental Figure 1

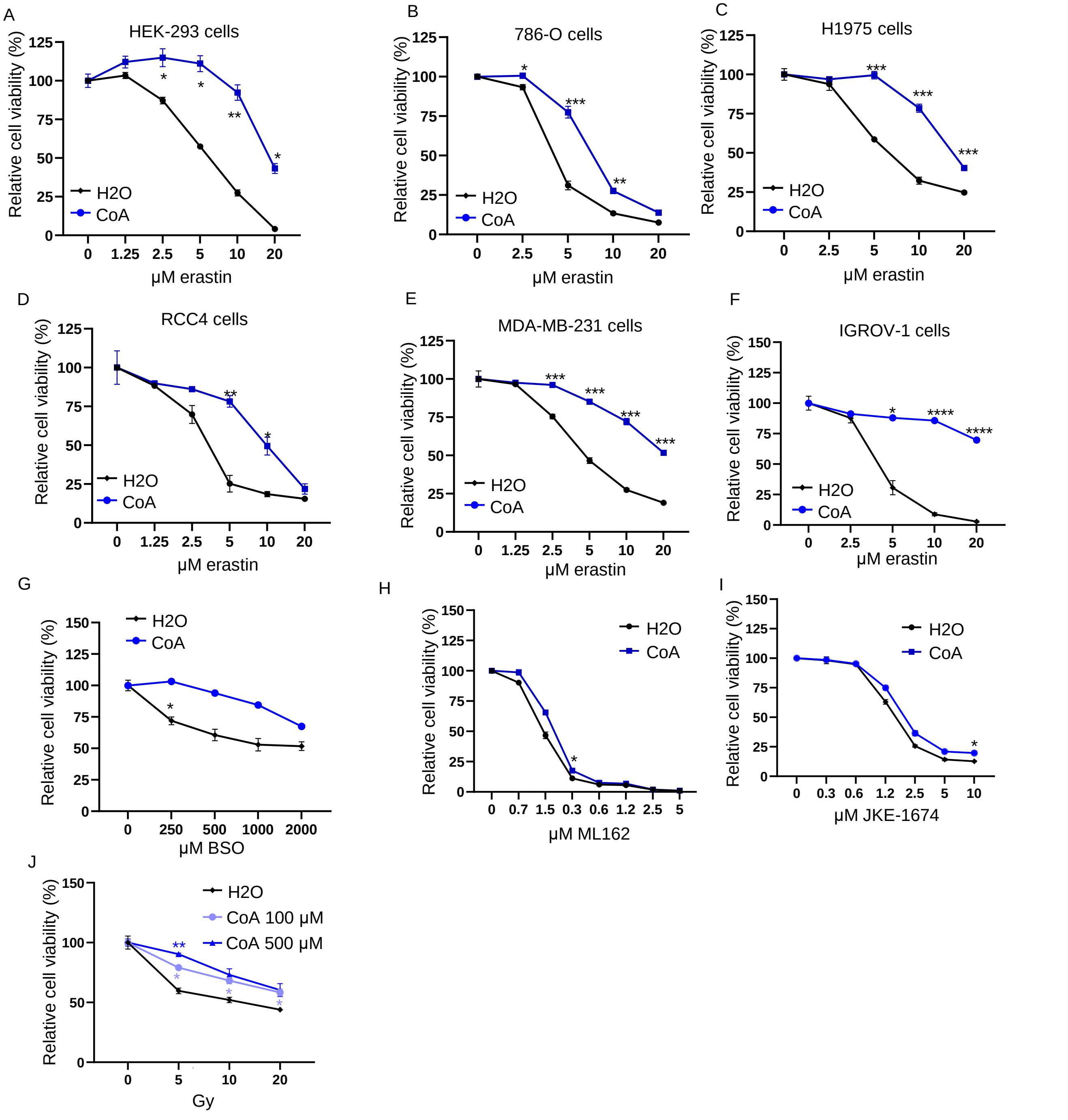

Supplemental Figure 1 The effects of CoA on the ferroptosis of various cancer cell lines induced by different FINs

(**A-F**) HEK-293 (**A**), 786-O (**B**), H1975 (**C**), RCC4 (**D**), MDA-MB-231 (**E**), and IGROV-1 cells (**F**), with CoA supplement (100 μM) were treated with varying doses of erastin for 18 hours. The cell viability was quantified by Cell-Titer Glo assay. (**G**) CoA (100 μM) inhibited class 1 FIN-induced ferroptosis in HT-1080 cells including BSO (3 days) (**H-I**) CoA (100 μM) failed to inhibit ferroptosis in HT-1080 cells induced by ML162 (class 2 FIN, 20 hours) (**H**) or JKE-1674 (class 2FIN, 20 hours). (**J**) HEK-293 cells with CoA supplement were exposed to various dose of radiation. Cell viability was measured 3 days after exposure. (**A-J**) Two-way ANOVA, Sidak's multiple comparisons, n = 3 independent biological replicates. $*p < 0.05$, $**p < 0.01$, $***p < 0.001$, and $****p < 0.0001$, The bars show standard error of the mean.

Lin_ Supplemental Figure 2

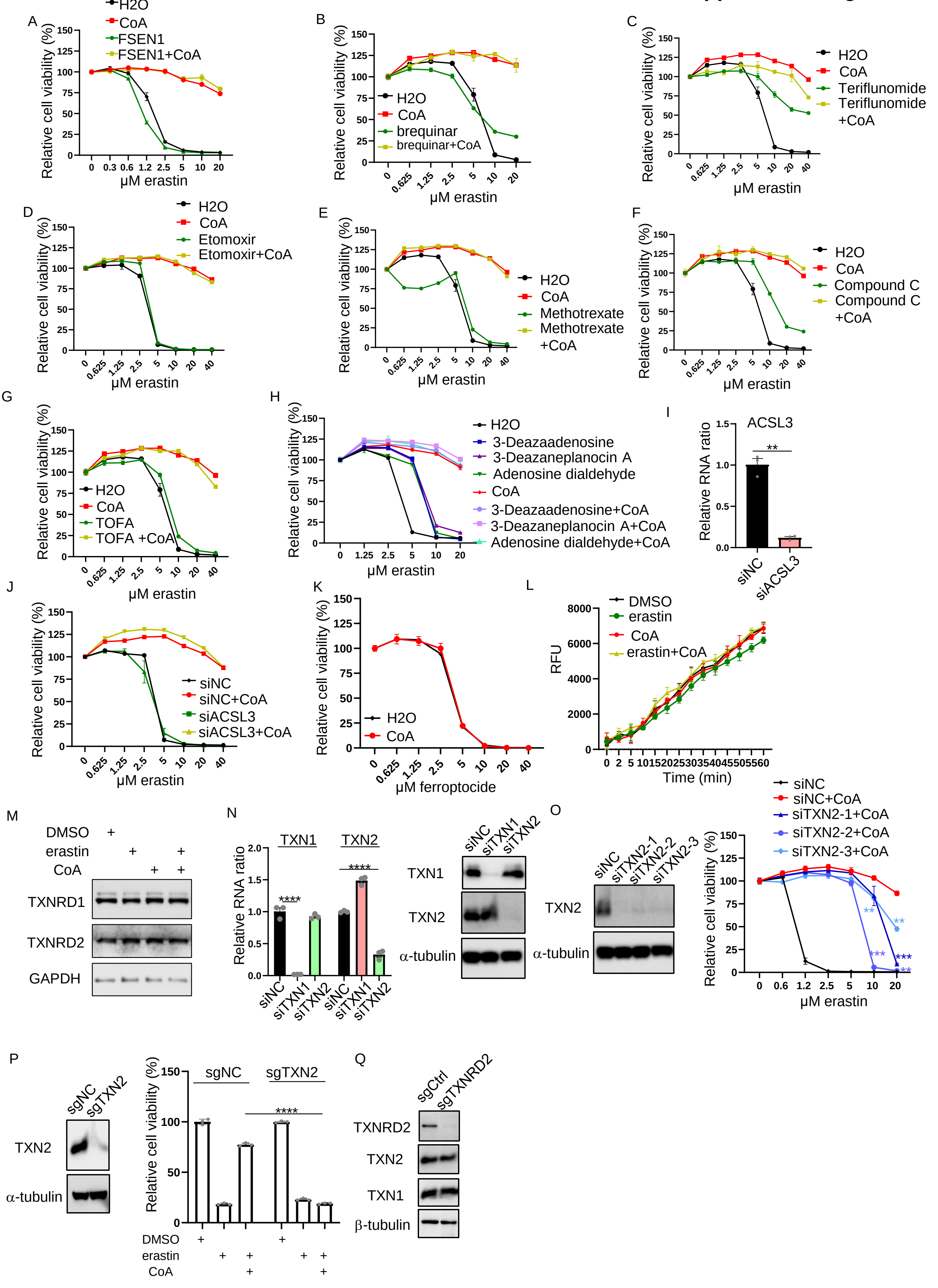

Supplemental Figure 2 The effects of different genetic and chemical inhibition of candidate targets on the CoA-mediated ferroptosis protection

(**A-J**) Screening for candidate inhibitors or siRNAs that can abolish CoA-mediated ferroptosis inhibition. FSP1 inhibitor (FSEN1, 2.5 μM) (**A**), DHODH inhibitor (brequinar, 500 μM) (**B**), DHODH inhibitor (Teriflunomide, 500 μM) (**C**), β-oxidation inhibitor (Etomoxir, 5uM) (**D**), dihydrofolate reductase inhibitor (Methotrexate, 2 μM) (**E**), AMPK inhibitor (compound C, 10 μM) (**F**), ACC inhibitor (TOFA, 25 μM) (**G**), S-adenosyl homocysteine hydrolase inhibitors (3-Deazaadenosine 10 μM, 3-Deazaneplanocin A 10 μM, Adenosine dialdehyde 10 μM) (**H**), siRNA knockdown of ACSL3 (**I-J**), were treated individually or in combination with CoA (100 μM) upon erastin treatment for 20 hours. The cell viability was quantified by Cell-Titer Glo assay. (**B, C, E, F, G**) were also performed at the same time. Each group has the same control (H2O) and CoA-treated samples. (**K**) CoA failed to rescue ferroptocide-induced cell death. HT-1080 cells with CoA (100 μM) were treated with 20 hours of various doses of ferroptocide and cell viability was measured by Cell-Titer Glo assay. (**L**) Individual or combination treatment of erastin (1.25 μM) and CoA (100 μM) for 16 hours did not alter thioredoxin activity in HT-1080 cell lysates. (**M**) Western blots showed that the treatment of erastin (1.25 μM, 16 hours) and CoA (100 μM), either alone or in combination, did not alter TXNRD1 and TXNRD2 protein levels.

(**N**) Validation of TXN1 and TXN2 siRNA knockdown at both RNA and protein levels in HT-1080 cells. (**O**) Individual siRNAs targeting three different regions of TXN2 with validation by WB abolished CoA (100 μM)-mediated ferroptosis protection against various doses of erastin for 20 hours. (**P**) TXN2 knockout in HT-1080 cells abolished the CoA (100 μM)-mediated ferroptosis protection against erastin (5 μM, 20 hours). (**Q**) TXNRD2 knockout did not alter TXN1 or TXN2 expression. (**N,W**) One-way ANOVA, Tukey’s multiple comparisons (**N,O,P**) Two-way ANOVA, Sidak’s multiple comparisons, n = 3 independent biological replicates. (**I**)Unpaired t-test. $*p < 0.05$, $**p < 0.01$, $***p < 0.001$, and $****p < 0.0001$, The bars show standard error of the mean.

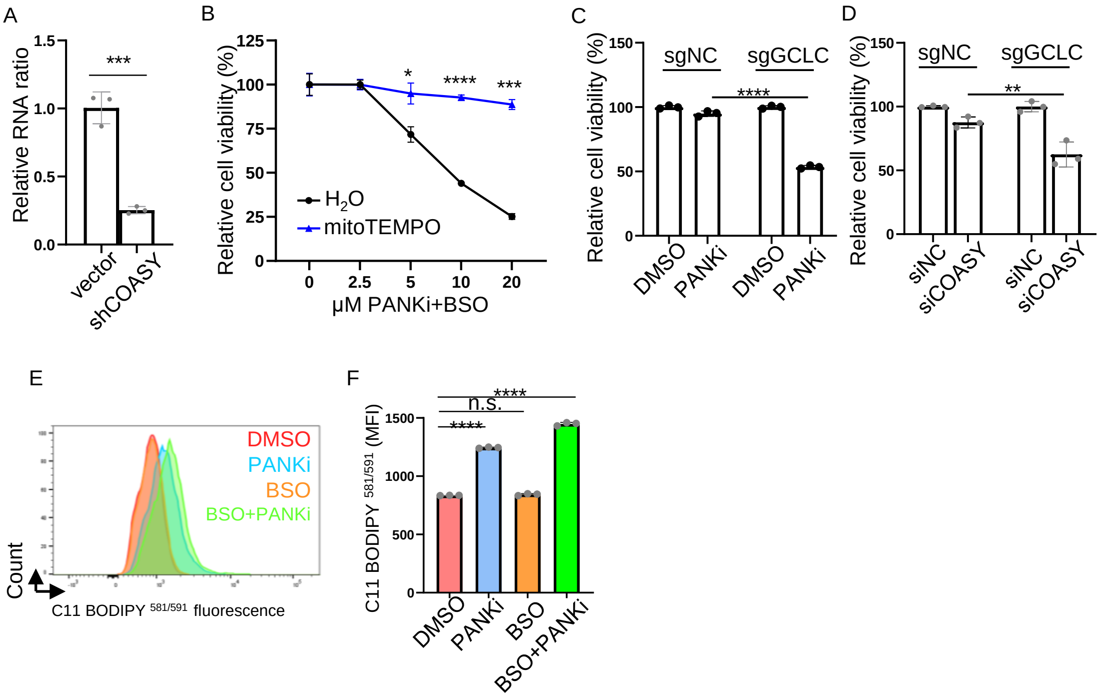

Supplemental Figure 3 Combined inhibition of glutathione and CoA synthesis lead to synthetic lethality by both pharmacological or genetic approach.
(**A**) Validation of COASY shRNA knockdown by qRT-PCR in HT-1080 cells. (**B**) mitoTEMPO (10 µM) abolished the cell death triggered by the combination of various dose of PANKi with BSO (300 µM) in HT-1080 cells for 24 hours. (**C**) GCLC knockout in HT-1080 cells synergized with PANKi (5 µM, 24 hours) to trigger cell death. (**D**) GCLC knockout in HT-1080 cells synergized with COASY knockdown (72 hours) to decrease cell viability as determined by crystal violet staining. (**E-F**) PANKi, but not BSO, increased overall lipid peroxidation (PANKi 2.5 µM, BSO 1 mM, 18 hours) in HT-1080 cells as determined by C11 BODIPY $^{581/591}$ staining (**E**) and the quantification of MFI (**F**). (**F**) One-way ANOVA, Tukey's multiple comparisons (**B,C,D**) Two-way ANOVA, Sidak's multiple comparisons, n = 3 independent biological replicates. (**A**)Unpaired t-test. $*p < 0.05$, $**p < 0.01$, $***p < 0.001$, and $****p < 0.0001$, The bars show standard error of the mean.

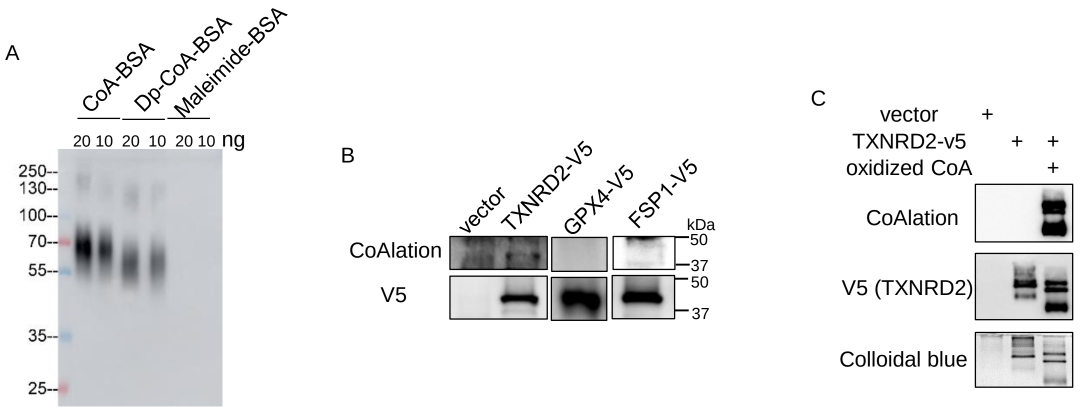

Supplemental Figure 4 Characterization of the TXNRD2 CoAlation

(**A**) Validation of CoAlation antibody. CoA, 3’-dephospho-CoA (Dp-CoA), or maleimide were conjugated to BSA and resolved on non-reducing SDS PAGE with 20 and 10 ng of protein for Western blots. (**B**) HT-1080 cells overexpression TXNRD2, FSP1, or GPX4 with V5 tag were purified by V5 agarose and blotted with CoAlation antibody. (**C**) CoAlation on TXNRD2 led to changes in the migration pattern. V5-tag purified TXNRD2 was treated with oxidized CoA for in vitro CoAlation, resolved on native PAGE, and stained with Colloidal blue or Western blots for CoAlation.

Lin_ Supplemental Figure 5

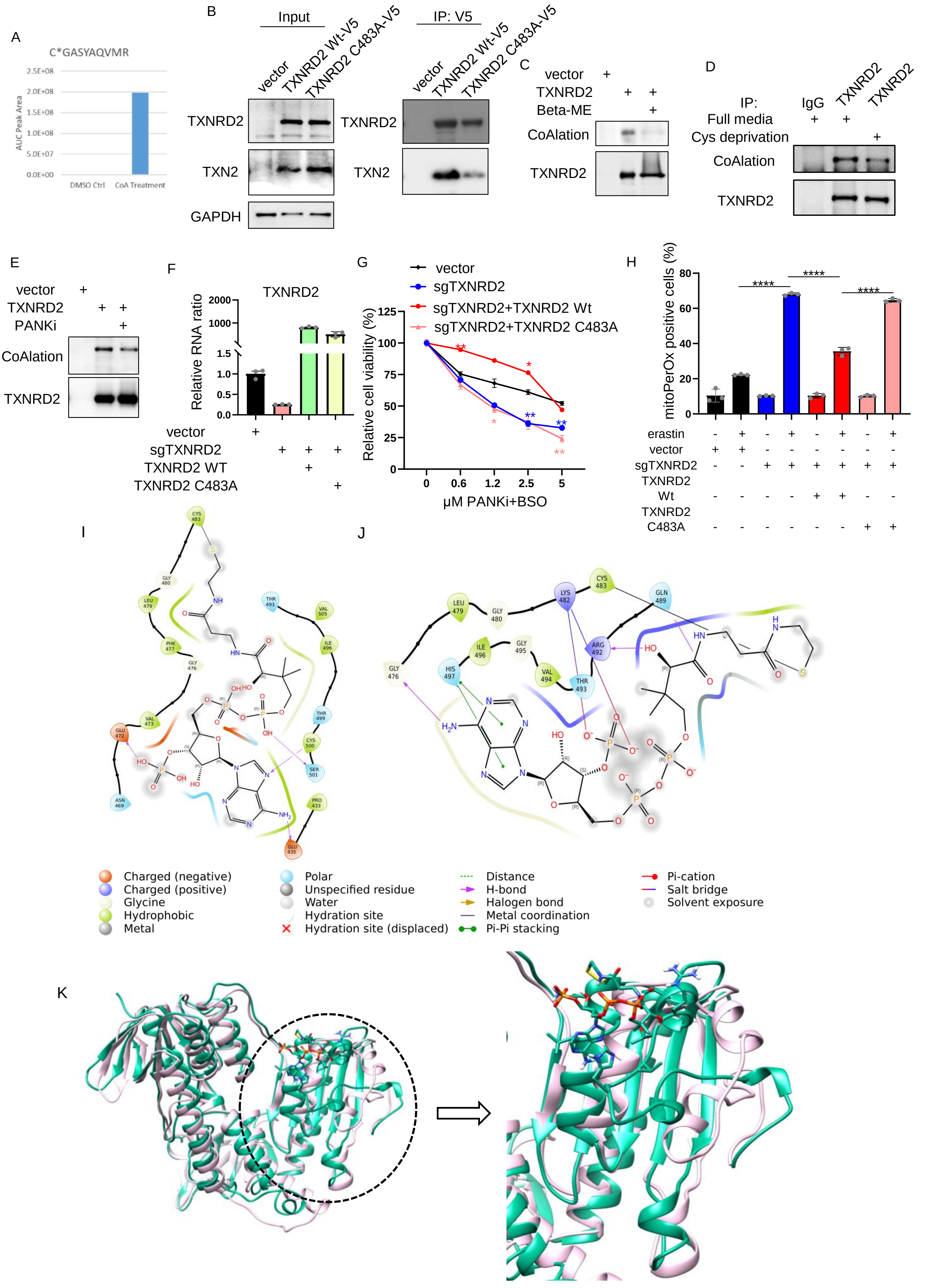

Supplemental Figure 5 Characterization of the TXNRD2 CoAlation

(**A**) Summed EIC peptide intensities from this peptide from each sample. (**B**) HT-1080 cells over-expressing TXNRD2 Wt or C483A with V5 tag were pulled down by V5 antibody for its interaction with TXN2. (**C**) Beta-mercaptoethanol (Beta-ME, 100 mM) treatment cleaved the disulfide bond and abolished CoAlation on purified TXNRD2 protein. (**D**) Protein lysates of IGROV-1 cells in full media or cystine deprivation (2 μM cystine, 24 hours) were pulled down by TXNRD2 antibody for endogenous levels of TXNRD2 and blotted for CoAlation. (**E**) PANKi treatment (5 μM, 20 hours) in HT-1080 cells reduced CoAlation of TXNRD2 protein. (**F**) Validation of TXNRD2 knockdown by TXNRD2 sgRNA and the over-expression of sgRNA-resistant TXNRD2 wild-type and C483A by Quantitative real-time PCR. (**G**) Cys-483 on TXNRD2 determines its function against ferroptosis induced by the combination of PANKi and BSO. HT-1080 cells were transduced with lentiviral sgRNA against TXNRD2 to knock out endogenous TXNRD2 expression and overexpressed with TXNRD2 wild-type or C483A mutant with synonymous mutation to avoid targeting by TXNRD2 sgRNA. These cell lines were treated with the combination of BSO (300 μM) and various concentrations of PANKi for Cell-Titer Glo assay. (**H**) Cys-483 on TXNRD2 determines its redox function against erastin-induced ferroptosis. HT-1080 cells were transduced with lentiviral sgRNA against TXNRD2 to knock out endogenous TXNRD2 expression and overexpressed with TXNRD2 wild-type or C483A mutant with synonymous mutation to avoid targeting by TXNRD2 sgRNA. These cell lines were treated with erastin (2 μM, 18 hours) for mitoPerOx staining. (**I-J**) 2D interaction diagram of the TXNRD2-CoA complex in docked pose (**I**) and 250ns MD snapshot (**J**). (**K**) Structural superimposition of the initial (pink) and 250 ns MD snapshot (green) of the TXNRD2-CoA complex. The C-terminal region, Glu412 to Thr518 is magnified. (**H**) One-way ANOVA, Tukey's multiple comparisons (**G**) Two-way ANOVA, Sidak's multiple comparisons, $n = 3$ independent biological replicates. $^{*}p < 0.05$, $^{**}p < 0.01$, $^{***}p < 0.001$, and $^{****}p < 0.0001$, The bars show standard error of the mean.

Lin_ Supplemental Figure 6

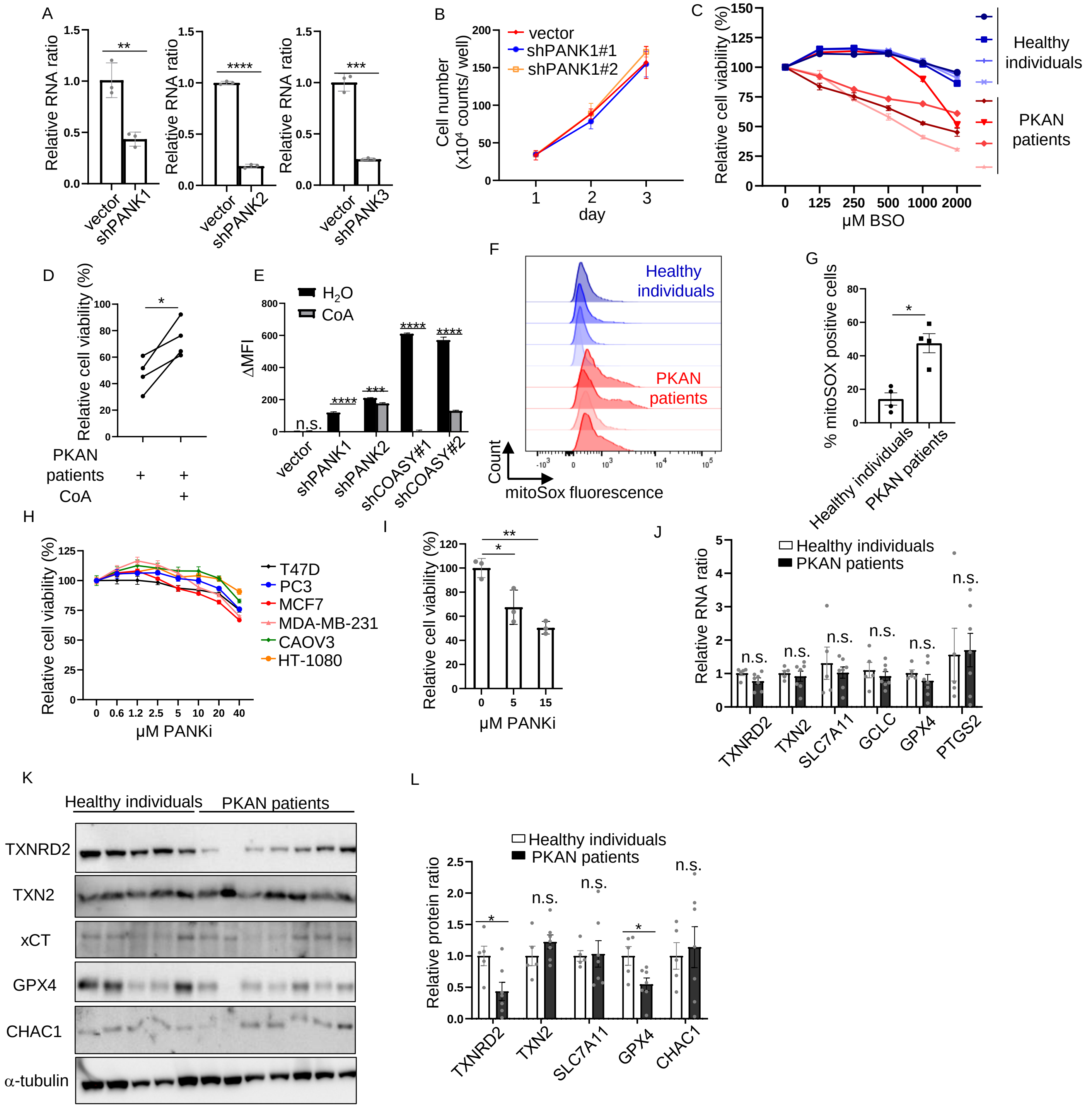

Supplemental Figure 6 The characterization of PANK as well as PKAN fibroblasts

(**A**) Validation of PANK1, PANK2, and PANK3 shRNA knockdown in HT-1080 cells by RT-PCR. (**B**) PANK1 knockdown did not alter cell proliferation rate. (**C**)The fibroblasts from PKAN patients are sensitive to BSO treatments. The fibroblasts from healthy individuals and PKAN patients were treated with an increasing dose of BSO for 2 days for Cell-Titer Glo assay. (**D**) CoA treatment rescued the PKAN fibroblasts from BSO-induced cell death. Within the same experiment in (**C**), PKAN fibroblasts with BSO were combined with CoA treatment and quantified Cell viability by Cell-Titer Glo assay. (**E**) The increase in mitochondrial lipid peroxidation by *PANK1*, *PANK2*, and *COASY* knockdown in HT-1080 cells was abolished by CoA supplement (100 µM, 20 hours). (**F**) PKAN fibroblasts, when compared with healthy fibroblasts, showed elevated mitochondrial ROS. Four pairs of fibroblasts from healthy individuals and PKAN patients were stained with the sensor of mitochondrial ROS (mitoSox). (**G**) The quantification of % mitochondrial ROS positive cells in (**F**). (**H)** PANKi treatment (72 hours) did not trigger obvious cell death in the six indicated cancer cell lines. (**I**) PANKi treatment (48 hours) induced cell death in N27 rat dopaminergic neural cell line. (**J**) The fibroblasts of PKAN patients showed no obvious changes of genes involving in mitochondrial thioredoxin system and ferroptosis markers at RNA levels as determined by qPCR. (**K**) Western blots showed that TXNRD2 and GPX4 were lower in the fibroblasts of PKAN patients. (**L**) Quantification of band intensity by Image J in (**K**). (**I**) One-way ANOVA, Tukey's multiple comparisons (**B,C,E**) Two-way ANOVA, Sidak's multiple comparisons, n = 3 independent biological replicates. (**A,D,G,J,L**) Unpaired t-test. *$p < 0.05$, **$p < 0.01$, ***$p < 0.001$, and ****$p < 0.0001$, The bars show standard error of the mean.

Supplemental Table 1. Primer sequences for qPCR.

Gene	Primer orientation	Sequence
TXN	Forward	GTGAAGCAGATCGAGAGCAAG
TXN	Reverse	CGTGGCTGAGAAGTCAACTACTA
TXN2	Forward	CTGGTGGCCTGACTGTAACAC
TXN2	Reverse	TGACCACTCGGTCTTGAAAGT
TXNRD2	Forward	CGGCTTCGACCAGCAAATG
TXNRD2	Reverse	ACAGGACGGTGTCAAAGGTG
GAPDH	Forward	GAGTCAACGGATTTGGTCGT
GAPDH	Reverse	TTGATTTTGGAGGGATCTCG
PANK1	Forward	TGGAACGCTGGTTAAATTGGT
PANK1	Reverse	CCCAGTTTTCCCATAAGCAGTAT
PANK2	Forward	TTGACTCAGTCGGATTCAATGG
PANK2	Reverse	CAGAAGCAGAGGATACGGATTTT
PANK3	Forward	TTTTGGCCGAAGAGGGAACTT
PANK3	Reverse	TAGCACCGTCTGCAATGTTGA
COASY	Forward	CCTGGGTCATCGGGCCTAT
COASY	Reverse	GGCCACATAATGTCCGTGAGTAT
ACSL3	Forward	CTTTCTCACGGATGCCGCATTG
ACSL3	Reverse	CTGCTGCCATCAGTGTTGGTTTC
