# Supplementary material for: Coenzyme A protects against ferroptosis via CoAlation of mitochondrial thioredoxin reductase
Source: J Clin Invest. 2025 Jul 22;135(19):e190215. doi: 10.1172/JCI190215 (PMC12483566; doi:10.1172/JCI190215)

Full unedited blots for

Coenzyme A protects against ferroptosis via CoAlation of mitochondrial thioredoxin reductase

Chao-Chieh Lin[1,2], Yi-Tzu Lin[1,2], Ssu-Yu Chen[1,2], Yasaman Setayeshpour[1,2], Yubin Chen[1,2], Denise E. Dunn[3], Taylor Nguyen[3], Alexander A. Mestre[1,2,4], Adrija Banerjee[5], Lalitha Guruprasad[5], Erik J. Soderblom[6], Guo-Fang Zhang[7,8], Chen-Yong Lin[9], Valeriy Filonenko[10], Suh Young Jeong[11], Scott R. Floyd[3], Susan J. Hayflick[11,12], Ivan Gout[10,13], Jen-Tsan Chi[1, 2].

Corresponding author: jentsan.chi@duke.edu

Full unedited blot/gel for Figure 2D

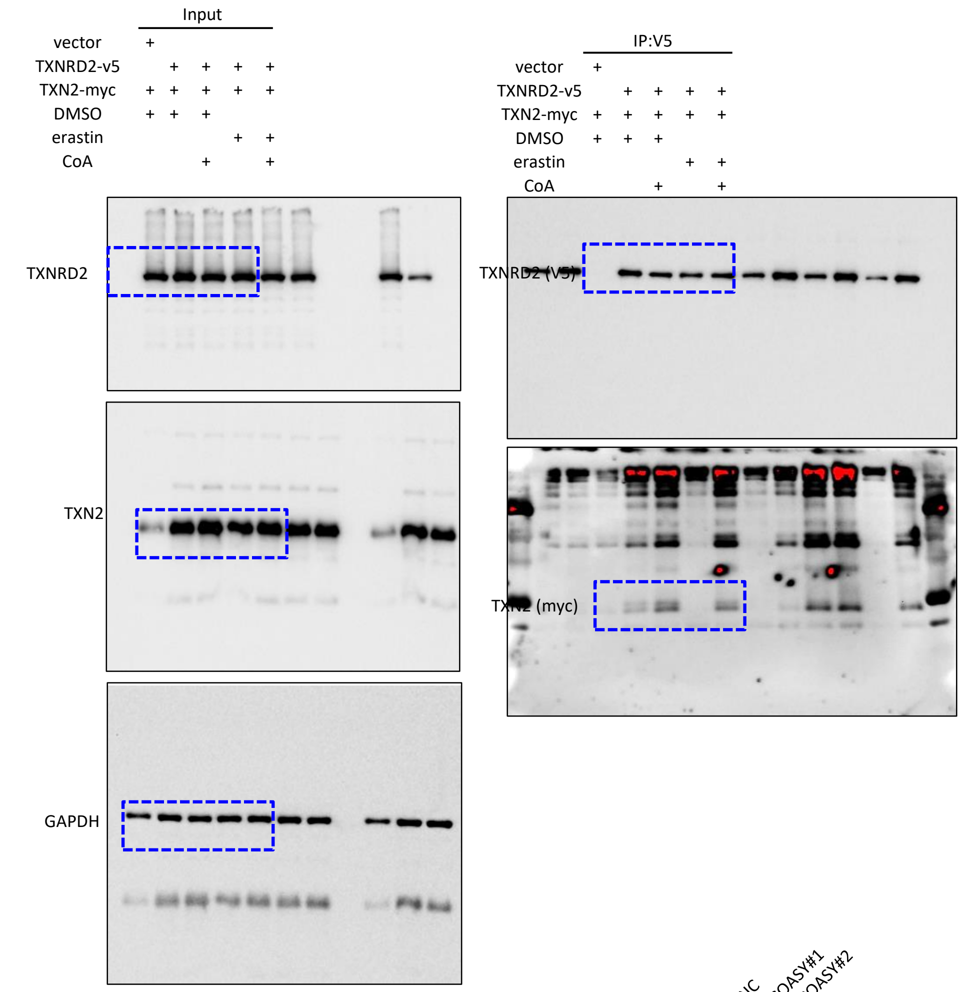

Full unedited blot/gel for Figure2E

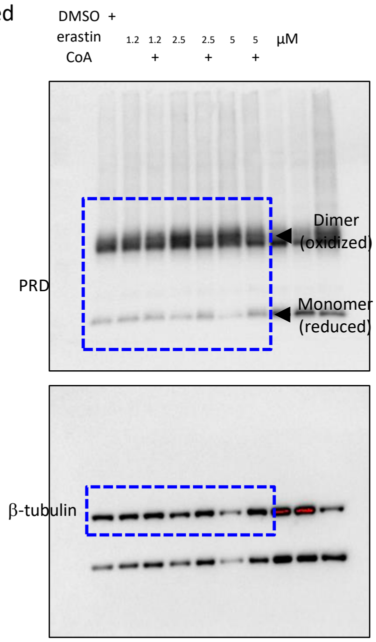

Full unedited blot/gel for Figure3A

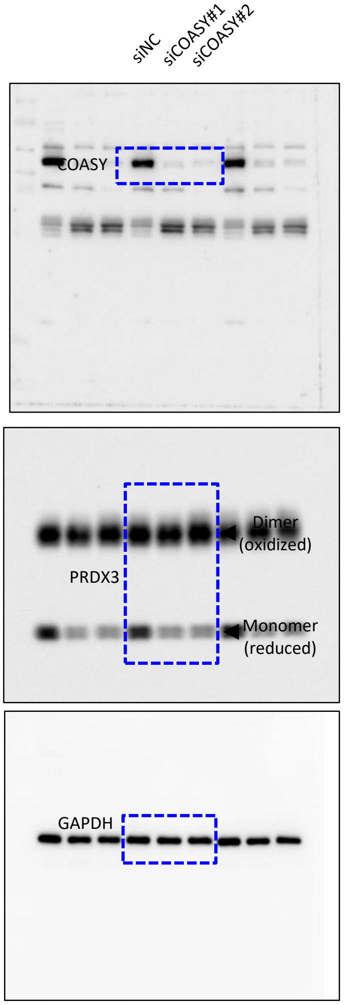

Full unedited blot/gel for Figure 4A

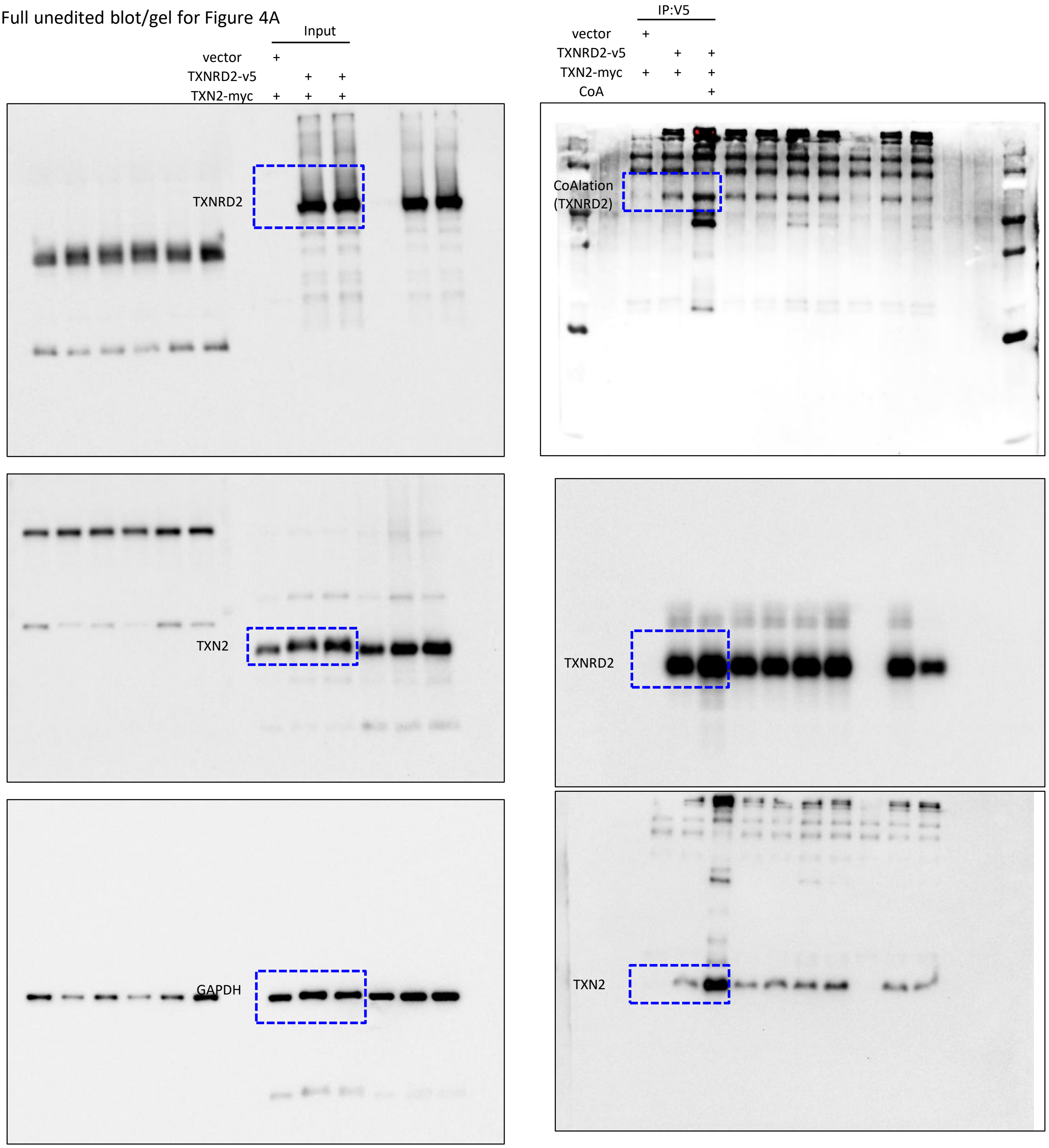

Full unedited blot/gel for Figure 4C

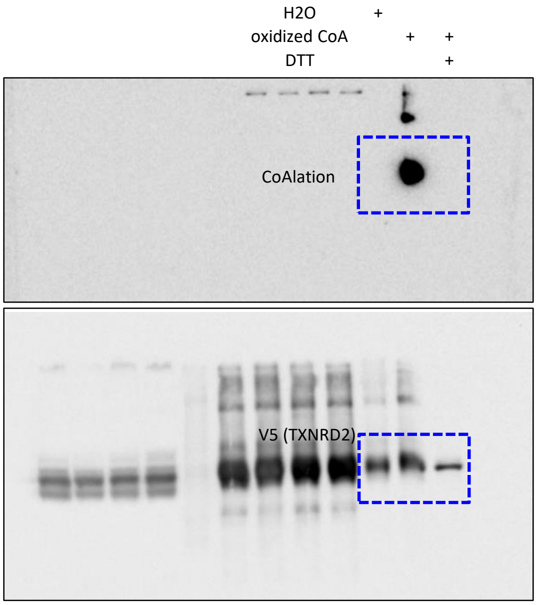

Full unedited blot/gel for Figure 5C

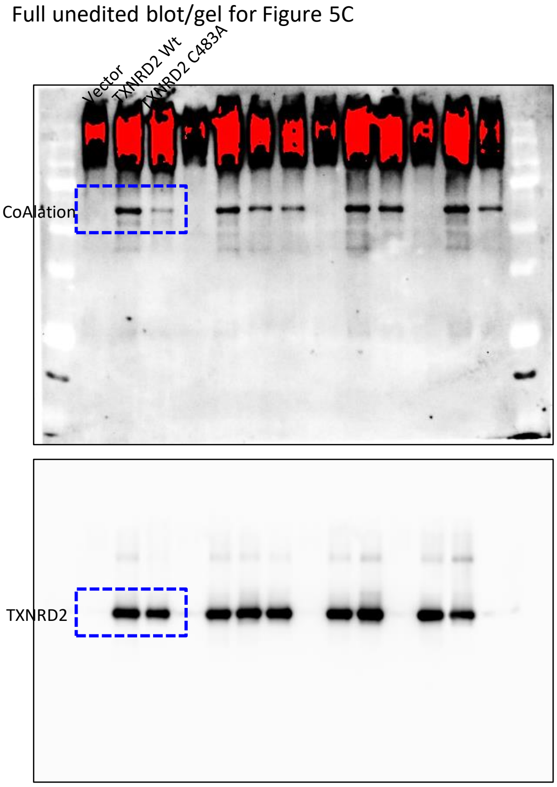

Full unedited blot/gel for Figure 5E

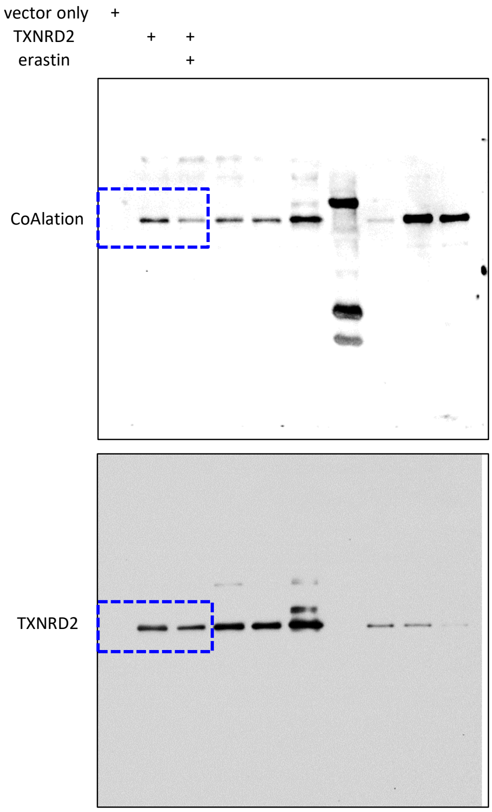

Full unedited blot/gel for Figure 6I

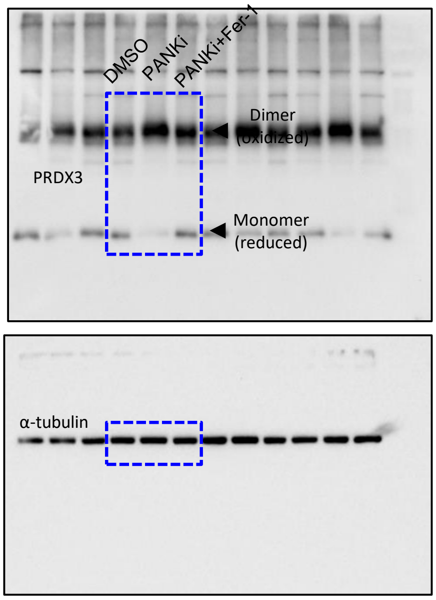

Full unedited blot/gel for Figure 6J

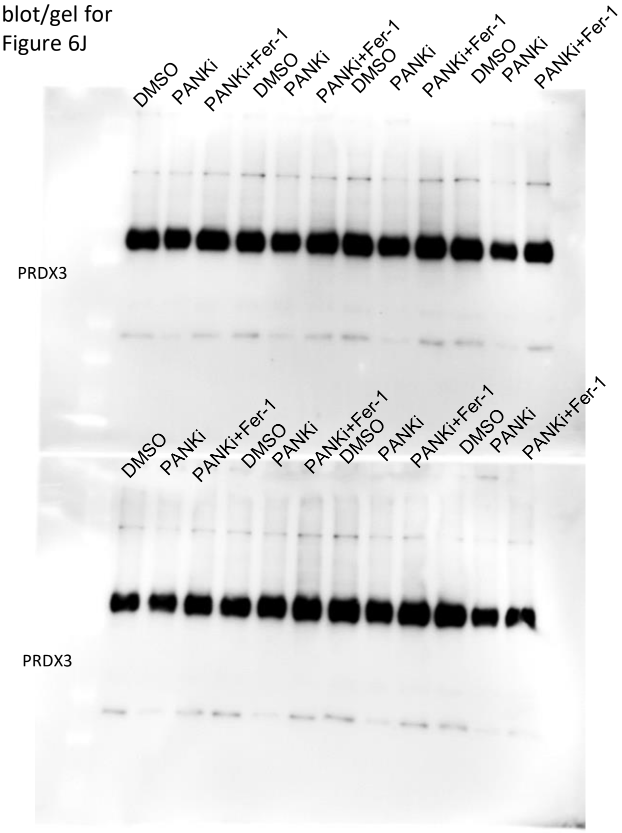

Full unedited blot/gel for Figure S2M

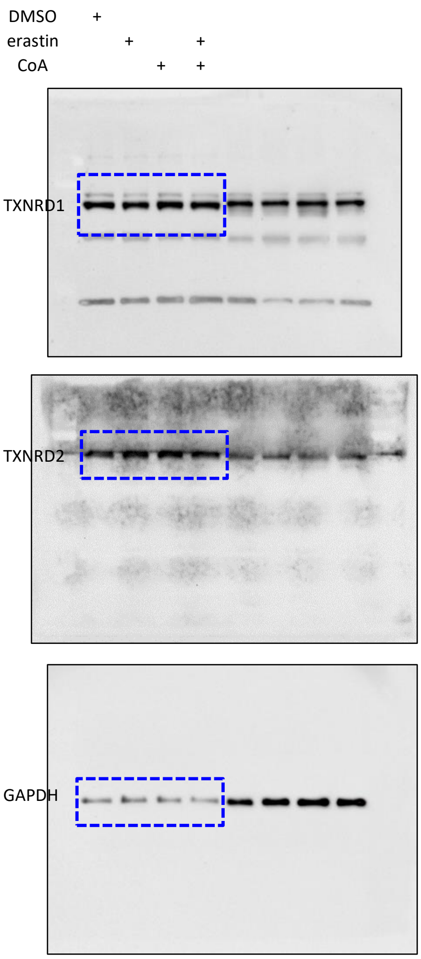

Full unedited blot/gel for Figure S2N

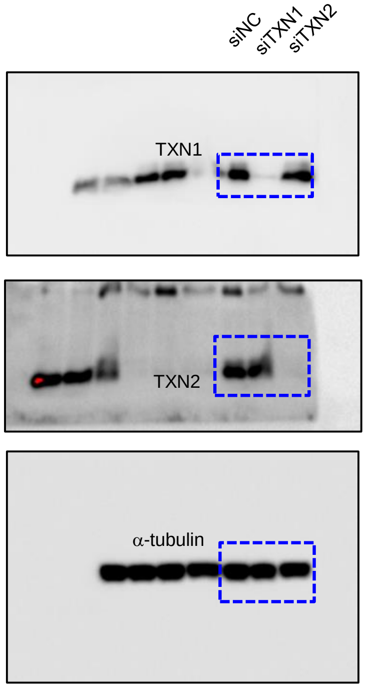

Full unedited blot/gel for Figure S2O

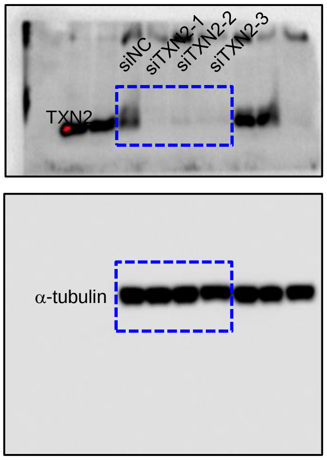

Full unedited blot/gel for Figure S2P

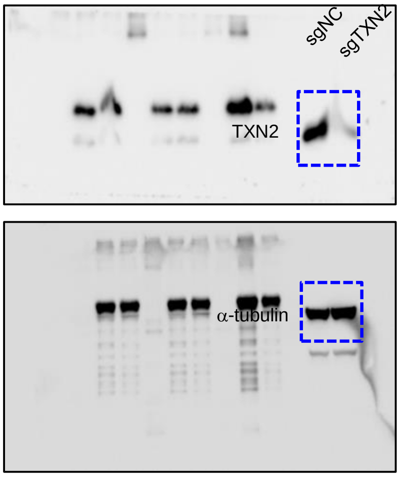

Full unedited blot/gel for Figure S2Q

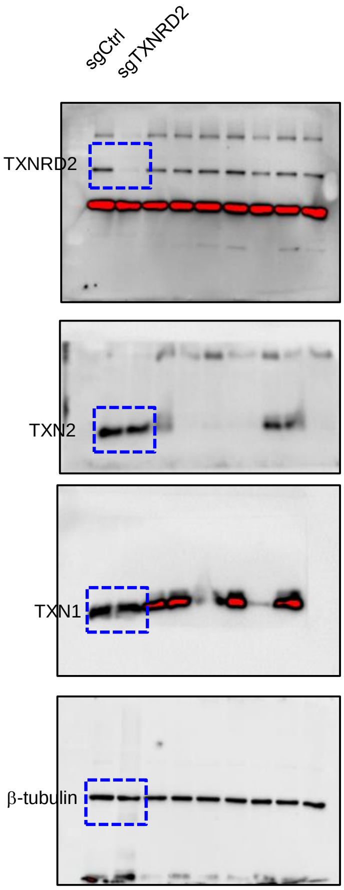

Full unedited blot/gel for Figure S4A

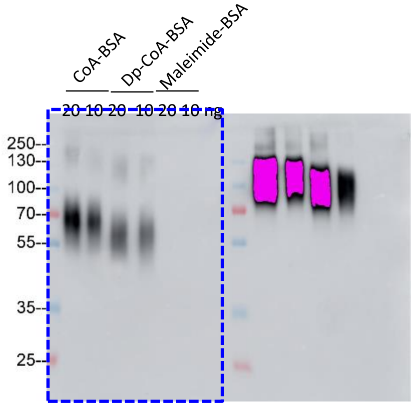

Full unedited blot/gel for Figure S4B

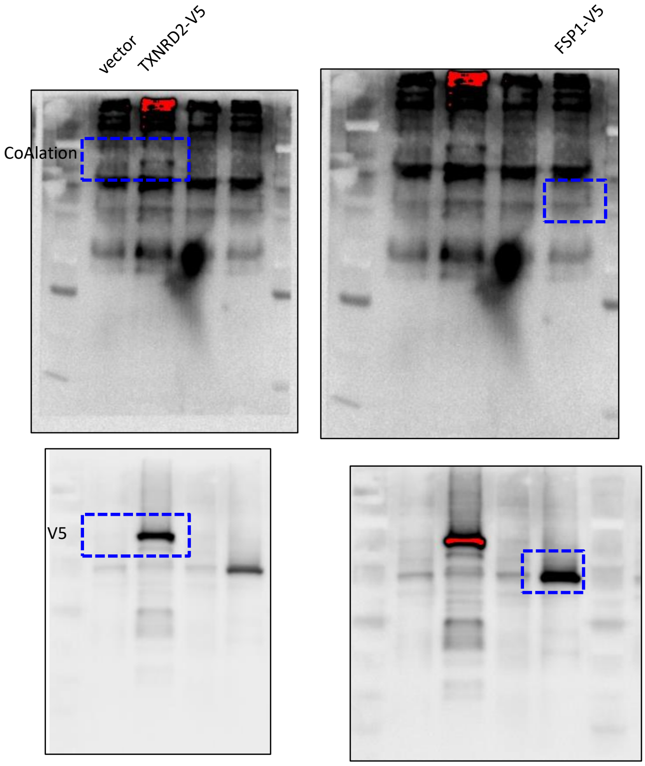

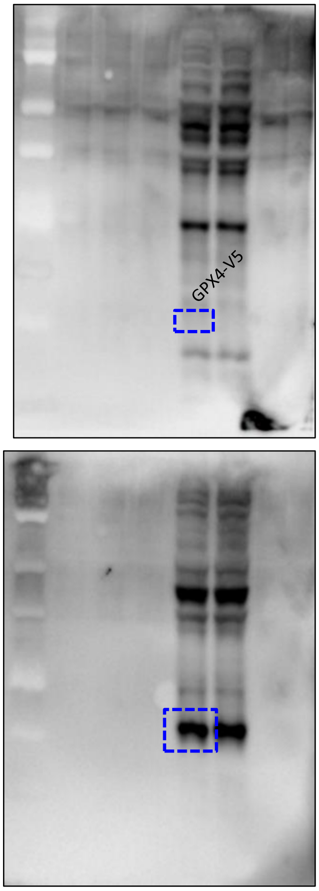

Full unedited blot/gel for Figure S4C

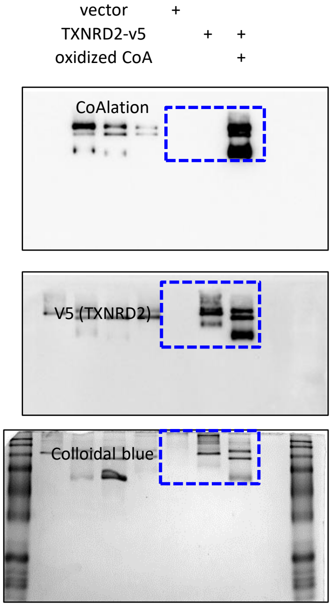

Full unedited blot/gel for Figure S5B

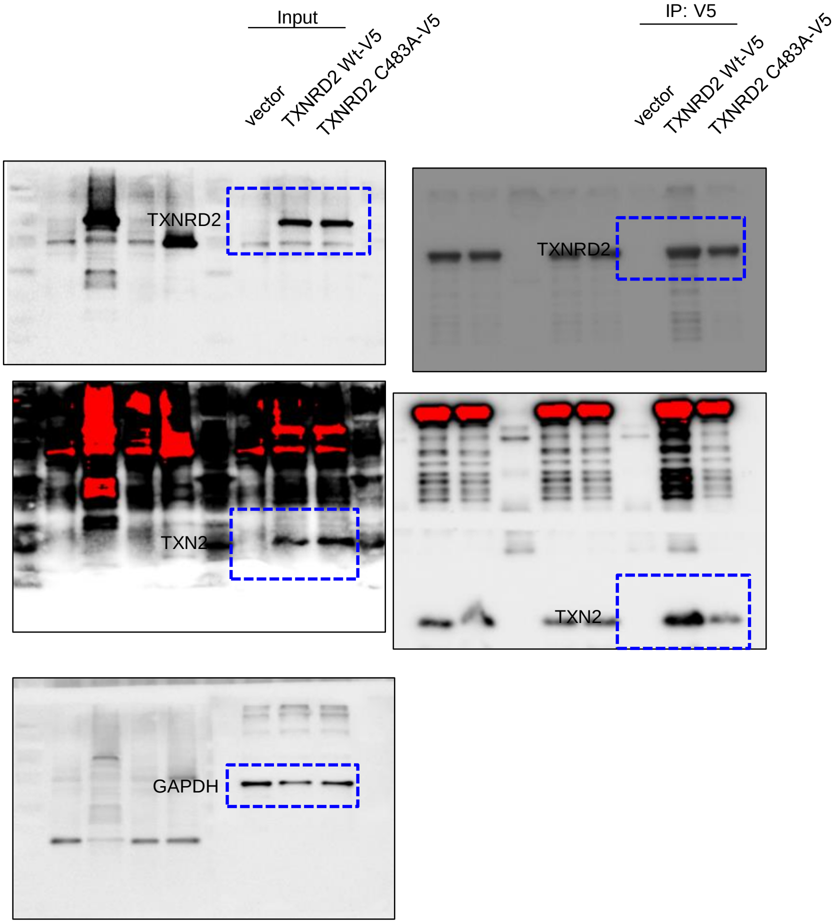

Full unedited blot/gel for Figure S5C

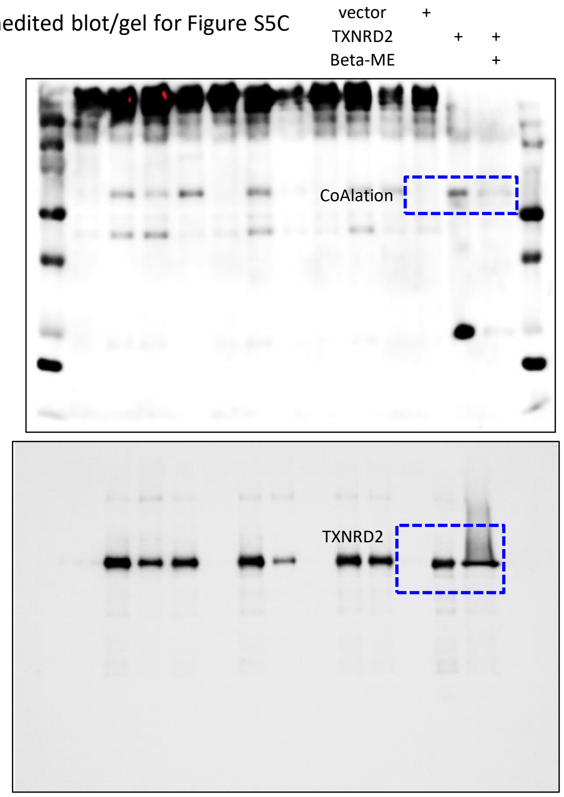

Full unedited blot/gel for Figure S5D

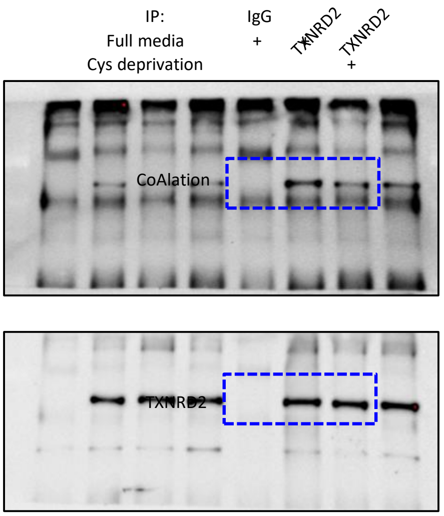

Full unedited blot/gel for Figure S5E

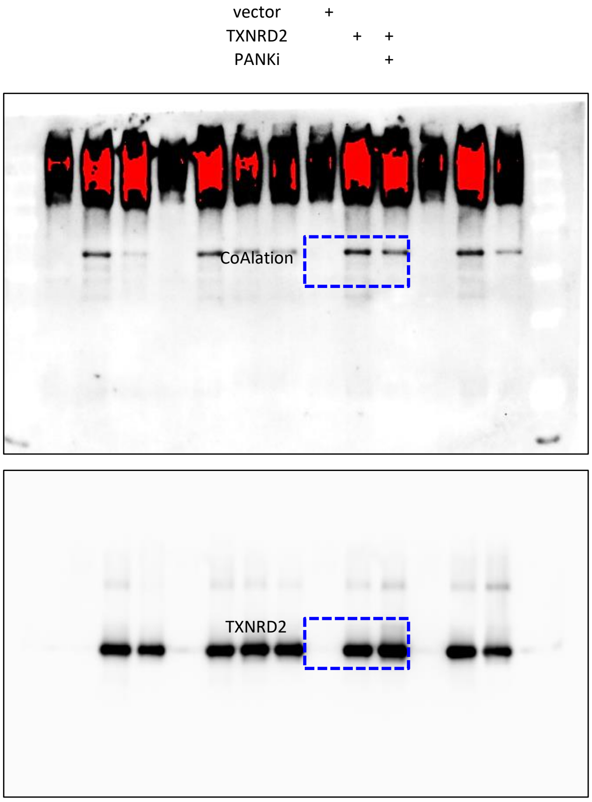

Full unedited blot/gel for Figure S6K

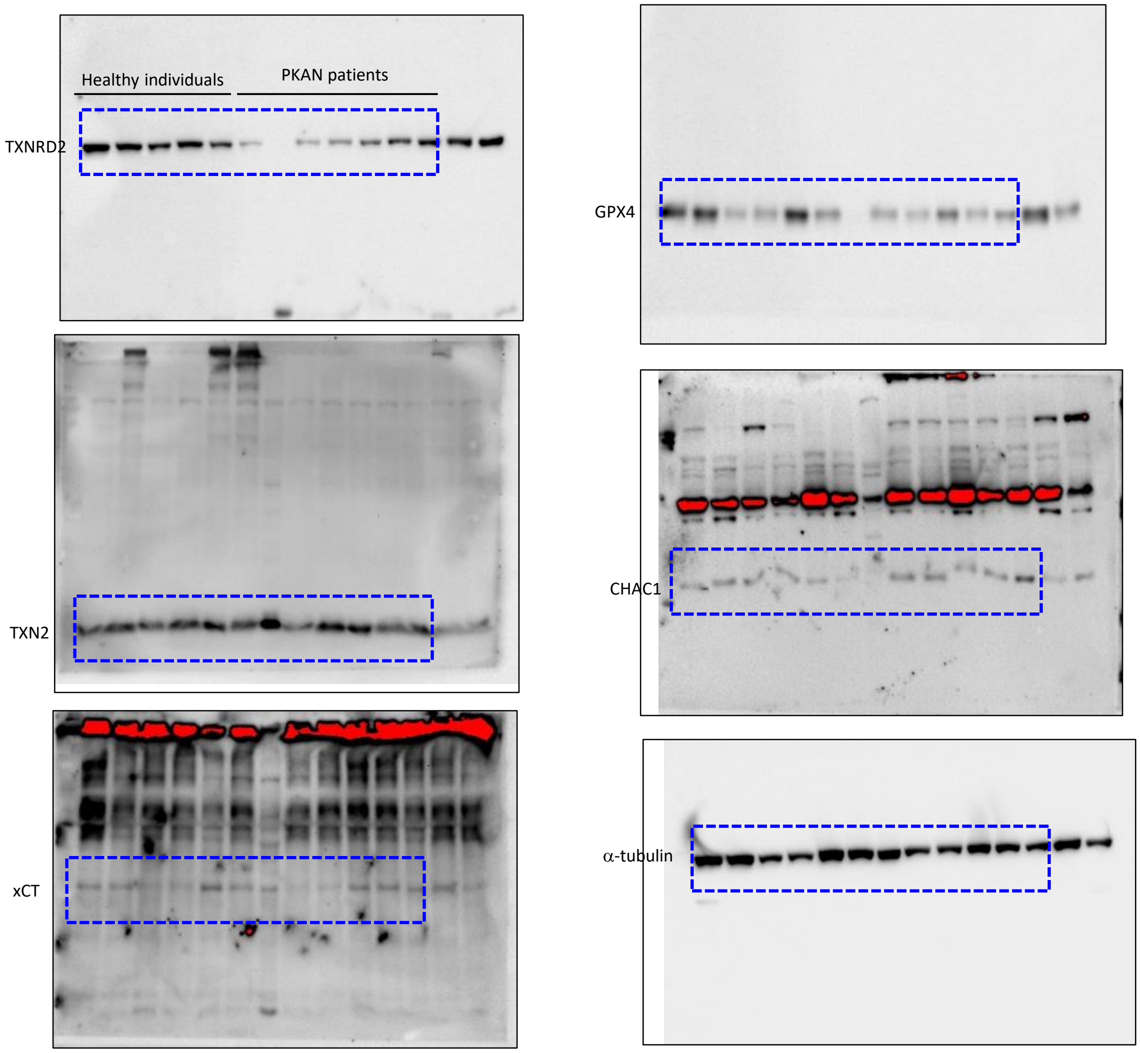

Supplement: Unedited blot and gel images [file jci-135-190215-s018.pdf]
